# 2′*-O-*ribose methylation levels of ribosomal RNA distinguish different types of growth arrest in human dermal fibroblasts

**DOI:** 10.1242/jcs.261930

**Published:** 2024-02-12

**Authors:** Guohuan Yang, Maximilian Schmid-Siegel, Clemens Heissenberger, Isabelle C. Kos-Braun, Martina Prechtl, Gabriel Meca-Laguna, Marta Rocha, Anja Wagner-Schrittwieser, Vera Pils, Barbara Meixner, Koray Tav, Markus Hengstschläger, Johannes Grillari, Martin Koš, Markus Schosserer

**Affiliations:** ^1^ Biochemistry Center (BZH), Heidelberg University, 69120 Heidelberg, Germany; ^2^ Institute of Molecular Biotechnology, Department of Biotechnology, University of Natural Resources and Life Sciences, 1190 Vienna, Austria; ^3^ Institute of Medical Genetics, Center for Pathobiochemistry and Genetics, Medical University of Vienna, 1090 Vienna, Austria; ^4^ Christian Doppler Laboratory for Skin Multimodal Imaging of Aging and Senescence, 1090 Vienna, Austria; ^5^ Ludwig Boltzmann Institute of Traumatology, 1200 Vienna, Austria; ^6^ Austrian Cluster for Tissue Regeneration, 1200 Vienna, Austria

**Keywords:** Cellular senescence, Epitranscriptome, 2′*-O-*methylation, rRNA, snoRNA, Ribosome biogenesis

## Abstract

The 2′*-O-*methylation (2′*-O-*Me) of ribosomal RNA (rRNA) shows plasticity that is potentially associated with cell phenotypes. We used RiboMeth-seq profiling to reveal growth arrest-specific 2′*-O-*Me patterns in primary human dermal fibroblasts from three different donors. We exposed cells to hydrogen peroxide to induce cellular senescence and to high cell densities to promote quiescence by contact inhibition. We compared both modes of cell cycle arrest to proliferating cells and could indeed distinguish these conditions by their overall 2′*-O-*Me patterns. Methylation levels at a small fraction of sites showed plasticity and correlated with the expression of specific small nucleolar RNAs (snoRNAs) but not with expression of fibrillarin. Moreover, we observed subtle senescence-associated alterations in ribosome biogenesis. Knockdown of the snoRNA SNORD87, which acts as a guide for modification of a hypermethylated position in non-proliferating cells, was sufficient to boost cell proliferation. Conversely, depletion of SNORD88A, SNORD88B and SNORD88C, which act as guides for modification of a hypomethylated site, caused decreased proliferation without affecting global protein synthesis or apoptosis. Taken together, our findings provide evidence that rRNA modifications can be used to distinguish and potentially influence specific growth phenotypes of primary cells.

## Teaser

**Highlighted Article:** RiboMeth-seq profiling of primary human dermal fibroblasts reveals growth arrest-specific patterns of rRNA 2′*-O-*methylation, which can distinguish and potentially influence specific growth phenotypes.

## INTRODUCTION

During the lifetime of an organism, its cells undergo multiple transient and permanent changes in response to various external and internal stimuli, including different types of growth arrest. In quiescence, cells become dormant and stop dividing. Quiescence is fully reversible and is characteristic of adult stem and progenitor cells residing in most tissues. These cells exit quiescence and re-enter the cell cycle to replace injured cells or to promote tissue growth (Urbán and Cheung, 2021).

In contrast, in senescence, cells typically stop dividing permanently and undergo profound physiological changes. The accumulation of senescent cells contributes to biological aging, mainly due to the characteristic pro-inflammatory senescence-associated secretory phenotype (SASP) (Coppé et al., 2008). Eliminating senescent cells extends the healthy lifespan of mice (Baar et al., 2017; Baker et al., 2016; Xu et al., 2018). Additionally, senescent cells promote age-related diseases, including cognitive decline and fibrosis, among others, and their targeted elimination leads to improved outcomes (Amor et al., 2020; Lushchak et al., 2023). Thus, strategies to deplete senescent cells or block the SASP, referred to as ‘senotherapies’, offer novel therapeutic opportunities for a wide range of age-related diseases (Cavinato et al., 2021; Robbins et al., 2021; Tchkonia et al., 2013). Understanding the distinct features of proliferating, quiescent and senescent cells is essential to enhance the specificity of these approaches for promoting healthy aging and preventing age-related diseases.

Ribosomes represent a promising target for senotherapies, as delayed ribosomal RNA (rRNA) processing promotes induction of senescence by activation of p53 (also known as TP53) in response to oncogenic and replicative stresses (Nishimura et al., 2015), and pre-ribosome accumulation maintains the senescent state by engaging pRb (also known as RB1) in models of oncogene-induced and replicative senescence (Lessard et al., 2018). Importantly, cellular senescence is a highly heterogeneous phenotype that is strongly influenced by the senescence inducer and cell type, among other factors. Recently, Papaspyropoulos and colleagues have demonstrated that replication-, genotoxic stress- and oncogene-induced senescence commonly reduce translational efficiency, and that particular changes in translational regulation depend on the specific senescence inducer (Papaspyropoulos et al., 2023). Heterogeneity in the protein composition of ribosomes in senescent cells (Shi et al., 2017) could explain some of these differences. However, other ribosome constituents, such as the most common types of rRNA modifications, remain unexplored in this context (Wagner and Schosserer, 2022).

2′*-O-*methylation (2′*-O-*Me), which with 104 known sites is the most abundant modification of human rRNA, is carried out by ribonucleoprotein complexes of four conserved proteins, including the methyltransferase fibrillarin (FBL), and a small nucleolar RNA (snoRNA) that specifies the methylated site (Kiss, 2002). 2′*-O-*Me contributes to ribosome heterogeneity and affects translational activity (Erales et al., 2017; Krogh et al., 2016). Evidence for the plasticity of 2′*-O-*Me modification of rRNA is constantly accumulating, such as in response to stress (Erales et al., 2017), during development and differentiation (Ramachandran et al., 2020; Hebras et al., 2020; Häfner et al., 2023), and during cancer progression (Krogh et al., 2020; Marcel et al., 2020).

Here, we show that in human dermal fibroblasts (HDFs), 2′*-O-*Me of rRNA and ribosome biogenesis subtly change in quiescence and senescence compared to that in proliferating cells. Moreover, depletion of snoRNAs that guide methylation at two specific variable sites either promotes or reduces cell proliferation without affecting apoptosis and global protein synthesis.

## RESULTS AND DISCUSSION

### 2′*-O-*Me patterns can distinguish proliferating, quiescent and senescent HDFs

To explore whether rRNA modifications change in response to stable or reversible growth arrest, we induced senescence in three distinct HDF strains from different donors (Table S1) by repeated exposure to hydrogen peroxide over two weeks (Lämmermann et al., 2018). After an additional week of recovery, we confirmed the senescent phenotype of the cells by staining for senescence-associated β-galactosidase (SA-β-gal) activity and cell cycle arrest by the absence of 5-bromo-2′-deoxyuridine (BrdU) incorporation (Fig. S1). We compared the stress-induced premature senescent (SIPS) cells to contact-inhibited quiescent (Q) cells and proliferating (P) cells derived from the same donors and at similar passage numbers in all experiments.

**Fig. 1.**
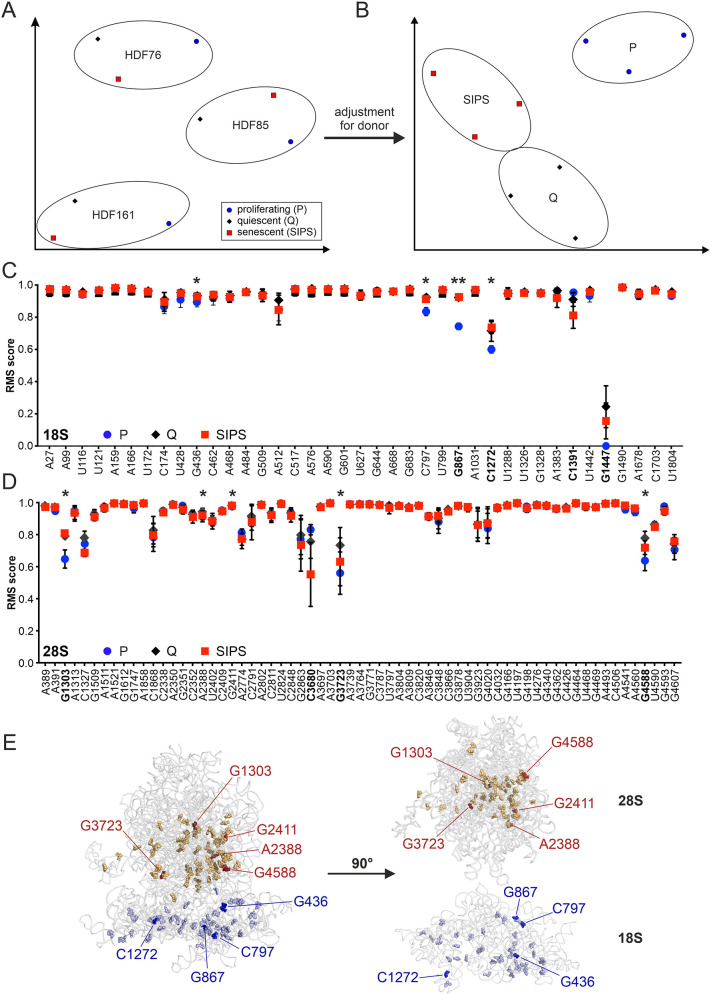
**Proliferating, quiescent and senescent HDFs can be distinguished by their rRNA 2′-*O*-Me pattern.** (A) Analysis of RMS profiling of proliferating, quiescent and senescent HDFs by *t*SNE revealed clustering according to the tissue donor. (B) After adjusting the multi-factor ANOVA statistical model for the factor ‘donor’, the samples clustered according to the cellular state in a *t*SNE plot. (C,D) Overview of 2′*-O-*methylated nucleotides determined by RMS in 18S (C) and 28S (D) rRNA. Mean RMS scores of three biological replicates (corresponding to the three tissue donors) are shown for each of the indicated cellular states. Sites selected for further investigation in Fig. 2 are printed in bold. Error bars represent the s.d. Statistically significant differences between the cellular states at specific sites were discovered using a multi-group ANOVA model after eliminating the factor ‘donor’ and setting a cutoff at *q*<0.1. α=0.05, *q<0.1, **q<0.01. (E) Known 2′*-O-*Me sites on human 18S and 28S rRNA were mapped on the human rRNA crystal structure (PDB: 6QZP; rRNA shown in gray, 18S sites shown in blue, 28S sites shown in yellow and red). Differentially methylated 18S and 28S rRNA sites are highlighted in dark blue and red, respectively.

To assess the methylation status of 104 known 2′*-O-*Me sites in the rRNA of SIPS cells, Q cells and P cells, we performed RiboMeth-seq (RMS) profiling (Krogh et al., 2016). Thereby, we assigned an RMS score (0=non-methylated, 1=fully methylated) to each modified position (Fig. 1). To reduce the dimensionality of the RMS data and visualize associations between the sample groups, we generated a *t*-distributed stochastic neighbor embedding (*t*SNE) plot revealing three distinct clusters corresponding to the three tissue donors (Fig. 1A). Thus, the donor of the cells appears to represent the main contributor to variability between samples, potentially masking differences between the cellular states. Indeed, after adjusting the multi-factor ANOVA statistical model for the factor ‘donor’, three distinct clusters of samples corresponding to SIPS cells, Q cells and P cells emerged (Fig. 1B). Notably, the two non-proliferative states, Q cells and SIPS cells, clustered closely together, whereas the cluster of P cells was more separated. Based on these data, we conclude that 2′*-O-*Me patterns can distinguish different cellular states, and that the most pronounced differences are between proliferative and non-proliferative cells.

**Fig. 2.**
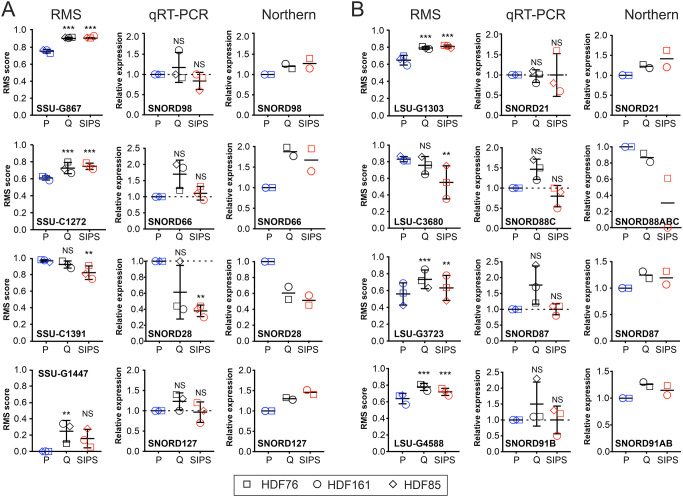
**2′*-O-*Me at selected sites correlates with snoRNAs guiding these modifications.** (A,B) RMS scores for selected variable 2′-*O*-Me sites in 18S (A) and 28S (B) rRNA, alongside expression levels of the snoRNAs guiding modification at each site, as assessed using qRT-PCR and northern blotting, for each of the indicated cellular states. Images of the northern blots used for quantification are presented in Fig. S2. *n*=3 biological replicates (corresponding to the three tissue donors) for RMS and qRT-PCR, and *n*=2 biological replicates for northern blots. Horizontal lines mark the mean, and error bars show the s.d. The statistical difference of RMS scores between groups was determined using a Tukey's range test following the multi-factor ANOVA (Fig. 1). For qRT-PCR data and northern blots, the expression was normalized to that of P cells, and two-tailed one-sample *t*-tests against an expected value of 1 were performed (only for qRT-PCR). α=0.05; **P<0.01; ***P<0.001; NS, not significant.

### A fraction of methylated sites show plasticity

Next, we inspected individual 2′*-O-*Me sites on the small ribosomal subunit (SSU) and the large ribosomal subunit (LSU) potentially underlying these global differences (Fig. 1C,D; Tables S2–S4). Whereas the majority of sites were highly methylated (RMS score>0.9), nine positions that were not fully methylated showed significant differences in methylation between the cellular states (SSU: G436, C797, G867, C1272; LSU: G1303, A2388, G2411, G3723, G4588; adjusted multi-factor ANOVA *q*<0.1). In line with our *t*SNE analysis (Fig. 1B), the most pronounced differences were again between P cells and the other two growth states (SSU: G436, C797, G867, C1272; LSU: G1303, G2411, G3723, G4588; Tukey's range test *P*<0.05; Table S2). Still, three sites also significantly differed in methylation between Q cells and SIPS cells (LSU: A2388, G3723, G4588; Tukey's range test *P*<0.05; Table S2), suggesting their specific association with cellular senescence. Interestingly, the average RMS scores for all sites were slightly higher in Q cells and SIPS cells than in P cells, with altered methylation sites predominantly occurring at G/C nucleotides.

Mapping the nine differently methylated sites to the three-dimensional structure of the human 80S ribosome (Fig. 1E) revealed that they were close to functional regions, including the intersubunit bridges, the polypeptide exit tunnel, and the body and head of the SSU (Fig. 1E). However, positions in the vicinity of the peptidyl transferase center (PTC) were not variable, indicating their essential role in core ribosome function.

### snoRNA levels partially explain the observed changes in 2′*-O-*Me patterns

In a recent study, the expression levels of FBL have been found to affect 2′*-O-*Me at specific positions in the HCT116 human colon carcinoma cell line (Sharma et al., 2017). However, in our primary fibroblast model, the 2′*-O-*Me in Q cells, SIPS cells and P cells did not correlate with FBL mRNA and protein expression, which tended to be decreased in SIPS cells from all donors (Fig. S1D–F). Instead, methylation at eight selected hypomodified sites – four on the SSU and four on the LSU – correlated with the expression of the snoRNAs guiding their modification. Even small methylation changes were accompanied by comparable alterations in the snoRNA levels (Fig. 2A,B; Figs S2 and S3). The snoRNA expression correlated with expression of the respective host gene in some, but not all, cases (Fig. S4). Notably, levels of all snoRNAs for the hypomodified sites were low, suggesting that the observed hypomethylation might be due to the limiting amounts of the snoRNAs.

### Hydrogen peroxide-induced senescence alters rRNA processing

**Fig. 3.**
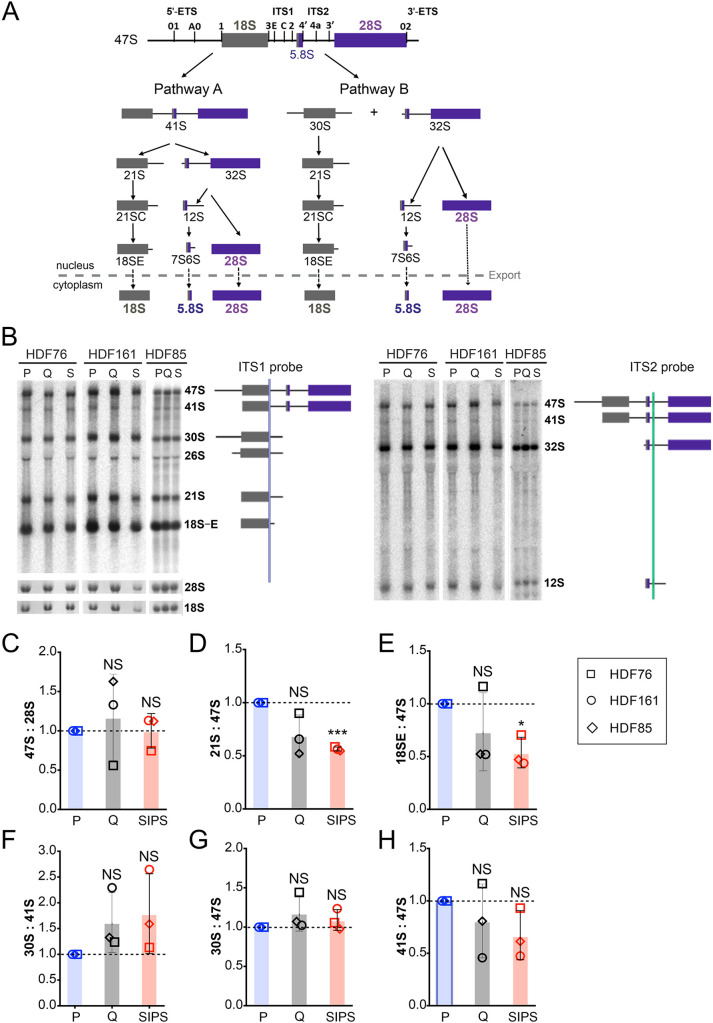
**Pre-rRNA processing is altered in HDFs undergoing hydrogen peroxide-induced senescence.** (A) Scheme of human pre-rRNA processing (based on the model by Mullineux and Lafontaine, 2012). Cleavage sites within the 47S rRNA external transcribed spacer (ETS) and internal transcribed spacer (ITS) sequences are indicated. (B) Northern blots of total RNA using ITS1 (left) or ITS2 (right) probes to detect different pre-rRNAs in HDFs from three different donors in the indicated cellular states. (C–H) Quantification of steady-state pre-rRNA levels expressed as ratios: (C) 47S:28S; (D) 21S:47S; (E) 18S-E:47S; (F) 30S:41S; (G) 30S:47S; (H) 41S:47S. Northern blot quantification data are shown as the mean of three biological replicates normalized to P cells. Error bars represent the s.d. Two-tailed one sample *t*-tests against an expected value of 1 were performed. α=0.05; *P<0.05; ***P<0.001; NS, not significant.

2′*-O-*Me is deposited during the early steps of ribosome biogenesis in the nucleus and is sensitive to subtle alterations in this process. Contrary to previous reports of delayed early rRNA processing in oncogene-induced and replicative senescence (Lessard et al., 2018; Nishimura et al., 2015), the 47S/45S pre-rRNA did not accumulate in SIPS cells, revealing that neither transcription nor early processing were affected (Fig. 3A–C). However, the relative levels of 21S and 18S-E pre-rRNAs decreased in SIPS cells, indicating an altered kinetics of the late SSU biogenesis (Fig. 3D,E). Intriguingly, Q cells and SIPS cells showed a slight preference for the rRNA processing pathway B, represented by an increase of the 30S:41S pre-rRNA ratio (41S and 30S pre-rRNAs are produced only by pathway A or pathway B, respectively) (Fig. 3A,F–H). Interestingly, an analogous switch between two pre-rRNA processing pathways in response to stress has been described in yeast (Kos-Braun et al., 2017). It is plausible that alternative pathways can provide means to produce distinct ribosomes.

### SNORD87 and SNORD88A/B/C depletion affects cell proliferation

**Fig. 4.**
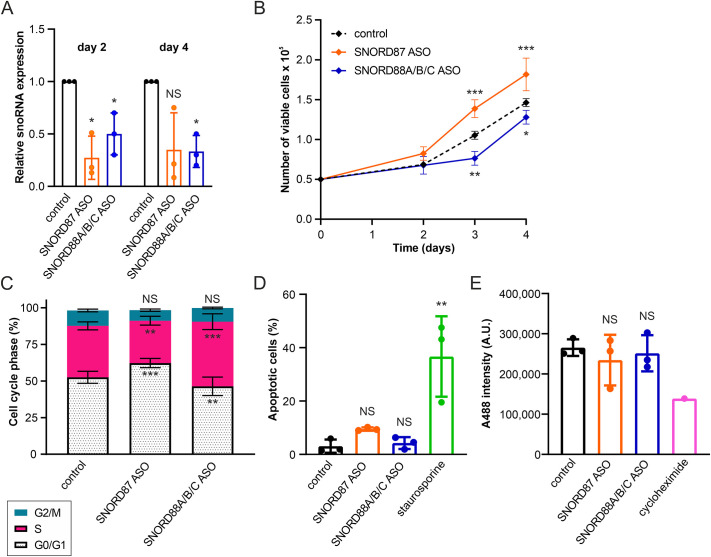
**Depletion of selected snoRNAs specifically promotes or inhibits cell proliferation.** HDF76 cells were transfected with ASOs specifically targeting SNORD87 or SNORD88A/B/C, as well as with a non-targeting scrambled control ASO (control). (A) qRT-PCR confirms knockdown of SNORD87 and SNORD88A/B/C 2 days and 4 days after transfection. The expression of SNORD87 and SNORD88C was normalized to that of 5.8S rRNA and plotted relative to expression in the control sample. SNORD88C is the predominant SNORD88 isoform in several adult tissues (Fafard-Couture et al., 2021), thus the observed reduction in SNORD88C expression upon SNORD88A/B/C ASO transfection was considered indicative of SNORD88A/B/C knockdown. (B) Knockdown of SNORD87 promotes, whereas knockdown of SNORD88A/B/C inhibits, proliferation of HDF76 cells, in comparison to HDF76 cells transfected with the control ASO. (C) Depletion of SNORD87 leads to an increase in the proportion of cells in G0/G1 phase and a decrease in the proportion of cells in S phase. In contrast, knockdown of SNORD88A/B/C leads to a decrease in the proportion of cells in G0/G1 phase and an increase in the proportion of cells in S phase. *n*=6. (D) Transfection of HDF76 cells with SNORD87 ASO or SNORD88A/B/C ASO does not induce apoptosis, as compared to cells transfected with the control ASO. Apoptosis was quantified as the percentage of annexin V-positive cells. Exposure to 1 µM staurosporine was used as a positive control. (E) Knockdown of SNORD87 or SNORD88A/B/C does not inhibit global protein synthesis, as compared to cells transfected with the control ASO. Global protein synthesis was quantified using an Alexa Fluor 488 (A488)-based OPP incorporation assay (A.U., arbitrary units). Cycloheximide treatment (50 µg/ml) was used as a specificity control (*n*=1). In A–E, the mean of three independent experiments is shown unless otherwise indicated. All error bars indicate the s.d. Statistical significance was tested by using two-tailed one-sample *t*-tests against an expected value of 1 (for A) or two-way ANOVA with Dunnett's post-hoc test (for B–E). α=0.05; *P<0.05; **P<0.01; ***P<0.001; NS, not significant.

Next, we wanted to test in HDFs whether the observed differences in 2′*-O-*Me between SIPS cells, Q cells and P cells are a consequence of other events or a prerequisite for the proliferative state. We focused on G3723 and C3680 in the LSU because these sites displayed the most considerable relative differences in methylation between Q cells and SIPS cells, with high donor variability, and had methylation levels that were oppositely correlated with cell proliferation despite being only 43 nucleotides apart. Interestingly, antisense oligonucleotide (ASO)-mediated knockdown of SNORD88A, SNORD88B and SNORD88C (SNORD88A/B/C) – the guides for methylation of LSU-C3680, which tended to be more methylated in P cells – indeed reduced proliferation. Conversely, knockdown of SNORD87 (the guide for methylation of LSU-G3723) led to increased proliferation, consistent with the reduced methylation of LSU-G3723 in P cells (Fig. 4A,B; Fig. S5). Cell cycle analysis using Hoechst 33342 staining revealed that SNORD88A/B/C depletion increased the fraction of cells in S phase and decreased the fraction of cells in G0 and/or G1 (G0/G1) phase, whereas transfection with the SNORD87 ASO resulted in the opposite effect (Fig. 4C; Fig. S6A). Surprisingly, we did not detect differences in apoptosis (Fig. 4D; Fig. S6B) or global protein synthesis (Fig. 4E) that might explain these observations. Taken together, these results indicate that differences in snoRNA expression between the P cells and the SIPS cells and Q cells are not just a side-effect but are more intrinsically linked to cell proliferation.

## Summary and conclusions

In summary, our 2′*-O-*Me profiling revealed distinct patterns of methylation in SIPS cells, Q cells and P cells that are consistent with the variable 2′*-O-*Me sites identified recently in cancer cells (Marcel et al., 2020). The most pronounced changes in 2′*-O-*Me were between proliferating and non-proliferating cells, although we also identified subtle alterations between SIPS cells and Q cells. Different tissue donors accounted for the majority of variability in 2′*-O-*Me. Still, our findings provide evidence that methylation patterns of rRNA provide, in principle, sufficient information to identify senescent cells despite their heterogeneity. Future studies will reveal whether 2′*-O-*Me signatures are specific enough to classify senescent cells, for instance, by artificial intelligence-driven deep learning approaches.

The catalytic core of the ribosome, the PTC, appears to be protected from changes, because most 2′*-O-*Me positions surrounding the PTC remained almost fully methylated. In contrast, SSU-C797 and SSU-G867, both of which are hypomethylated in P cells, are close to each other in the body of the SSU in the vicinity of bridge eB12, which consists of a salt bridge between eL19 (RPL19) and SSU-G909 and SSU-G910 (Kisly et al., 2016). LSU-C1303 is near uL4 (RPL4), contributing to the second constriction site of the polypeptide exit tunnel (Duc et al., 2019). LSU-G3723 is near the interaction layer of the SSU and LSU, the decoding center, and the B2a intersubunit bridge. This region is particularly interesting because it undergoes significant rearrangements to fine-tune the rotation of the ribosome during translation (Lomakin et al., 2017; Sulima et al., 2014).

Strikingly, several sites that we identified to be hypomodified in P cells also show lower modification levels in developing mouse tissues, relative to levels in adult tissues (Häfner et al., 2023; Hebras et al., 2020); in human embryonic stem cells, relative to levels in differentiated ectoderm (Häfner et al., 2023); in diffuse large B-cell lymphoma, relative to levels in reactive lymph nodes (Krogh et al., 2020); and in breast cancer cell lines (Marcel et al., 2020) – suggesting an inverse correlation between methylation of these sites and cell proliferation, cancer and pluripotency (Fig. S7).

Our results indicate that even small changes in snoRNA levels might directly affect the proliferative status of cells. Knockdown of SNORD87, a guide for a site that is hypomethylated in P cells, was sufficient to increase the proliferation of HDFs from two out of three tissue donors and to reduce the fraction of S-phase cells, suggesting a faster progression through the cell cycle. Surprisingly, this acceleration of cell proliferation was not accompanied by a detectable increase in global protein synthesis. Thus, methylation of LSU-G3723 might affect other functions of the ribosome, such as the recruitment of specific mRNAs for translation or translational fidelity, which could underlie the changes in cell proliferation. It is not uncommon for single RNA modifications to only subtly influence translation, as we have previously demonstrated for the two 5-methylcytosine (m^5^C) modifications on the LSU (Heissenberger et al., 2020; Schosserer et al., 2015). Similarly, knockout of SNORA24, a snoRNA guiding SSU pseudouridine (Ψ) deposition, does not alter polysome profiles and global protein synthesis but does affect the loading of specific tRNAs and translational fidelity. SNORA24 has been found to be required for oncogene-induced senescence and tumor suppression (McMahon et al., 2019).

Although our study shows that 2′*-O-*Me levels can distinguish different types of cellular growth arrest, it has certain limitations. We exclusively focused on 2′*-O-*Me, representing only one type and slightly less than half of all rRNA modifications. Moreover, only a few 2′*-O-*Me sites showed plasticity. Thus, including Ψ and base modifications in future experiments will likely improve the classification of different cellular states and identify additional sites specifically associated with cell proliferation and senescence. Similarly, we concentrated on HDFs and one inducer of cellular senescence and quiescence each. Further studies will be required to determine whether the discovered mechanisms are universal and whether the same sites are differentially modified in other cell types, in oncogene-induced or replicative senescence, and in serum-starvation-induced quiescence. Finally, whether the observed changes in 2′*-O-*Me patterns and the shift in ribosome processing pathways play an active part in regulating proliferation and senescence – for instance, by modulating specific mRNA translation – still remains to be tested.

## MATERIALS AND METHODS

### Cell culture

Primary human dermal fibroblasts (HDFs) from three healthy donors (HDF76, HDF85 and HDF161) were obtained from Evercyte GmbH (Vienna, Austria) and authenticated before freezing. Evercyte GmbH obtained documented ethical approval for skin cell isolation from local authorities and acquired informed consent from tissue donors. Details of all donors are listed in Table S1. Cells were cultured with DMEM/Ham's F-12 medium (1:1 mixture; F4815; Biochrom GmbH, Berlin, Germany) supplemented with 10% fetal calf serum (F7524; Sigma-Aldrich, St. Louis, MO, USA) and 4 mM L-glutamine (G7513; Sigma-Aldrich, St. Louis, MO, USA) under ambient oxygen and 5% CO_2_ at 37°C. Cells were regularly tested for mycoplasma contamination. Cell counting was performed using an automated Vi-CELL XR cell counter (Beckman Coulter, Brea, CA, USA).

Proliferating cells were passaged twice a week, and were detached by incubation with 0.1% trypsin and 0.02% EDTA at 37°C for 5 min, then split at ratios between 1:2 and 1:3 depending on cell type and growth rate. For the quiescent and SIPS states, cells were seeded at a density of 3500 cells/cm². Quiescent cells were then left to grow until contact inhibited (while exchanging medium once a week) in parallel to the SIPS cells being treated to induce senescence (see below).

### Stress-induced premature senescence by hydrogen peroxide exposure

SIPS was induced as previously described (Lämmermann et al., 2018). Due to differences in the growth behavior and replicative lifespan (Table S1), cells from different donors were seeded at the corresponding population doubling level (PD) at a density of 3500 cells/cm² and left to attach overnight. The following day, stress treatment was started. The 30% H_2_O_2_ stock solution (H1009; Sigma-Aldrich, St. Louis, MO, USA) was freshly diluted and added to the medium at a final concentration of 60–80 μM, depending on the donor and cell batch. After incubation at 37°C for 1 h, the medium was replaced. The hydrogen peroxide treatment was performed for 4 consecutive days, followed by a 2-day break, another 5 consecutive days of treatment, and finally, 5 or 6 days of recovery.

Induction of premature senescence was confirmed by the altered cellular morphology and SA-β-gal activity. Cell cycle arrest was assessed by BrdU incorporation.

### Senescence-associated β-galactosidase staining

SA-β-gal staining was performed as previously described (Dimri et al., 1995; Lämmermann et al., 2018). Ten images were captured for each well at 100× magnification, and positive cells were counted by a researcher who was unaware of the sample groups.

### 5-bromo-2′-deoxyuridine labeling

Cells were incubated in DMEM/Ham's medium supplemented with 10 μM BrdU (Sigma-Aldrich, St. Louis, MO, USA) for 24 h. After harvesting with trypsin, cells were fixed using ice-cold 70% ethanol for 1 h at 4°C. Denaturation of the DNA for 30 min with 2 M HCl and 1% Triton X-100 (Sigma-Aldrich, St. Louis, MO, USA) was performed, followed by neutralization with 0.1 M sodium borate, pH 8.5. Pellets were resuspended in reaction buffer (0.5% Tween 20 and 1% BSA in 1× PBS) containing anti-BrdU antibody (#347580; Becton, Dickinson and Company, Franklin Lakes, NJ, USA) at a 1:50 dilution and incubated for 30 min at room temperature. After washing with reaction buffer and staining with anti-mouse IgG FITC-conjugated secondary antibody (F-8264; Sigma-Aldrich, St. Louis, MO, USA) at a 1:500 dilution for 30 min, the pellet was washed with reaction buffer and resuspended in 1× PBS containing 2.5 μg/ml propidium iodide (PI) (Sigma-Aldrich, St. Louis, MO, USA). For compensation, cells were stained with either PI or BrdU alone. The analysis was performed by flow cytometry on a CytoFLEX S (Beckman Coulter, Brea, CA, USA).

### RNA extraction and purification

Cells were harvested, and total RNA was isolated using TRI Reagent (Sigma-Aldrich, St. Louis, MO, USA) following the manufacturer's protocol. Further purification of total RNA was achieved by washing the RNA pellet twice with 70% ethanol. The RNA concentration was measured with an ND-1000 NanoDrop spectrometer (Thermo Fisher Scientific, Waltham, MA, USA).

### Quantitative reverse transcription PCR

cDNA was synthesized from 50 ng total RNA using the High-Capacity cDNA Reverse Transcription Kit (Life Technologies, Waltham, MA, USA). Target gene expression levels were quantified using 5× HOT FIREPol EvaGreen qPCR Mix Plus (Solis BioDyne, Tartu, Estonia) on a Rotor-Gene Q cycler (Qiagen, Hilden, Germany). *GAPDH* was used as a housekeeping gene for the normalization of *FBL*.

As previously described, identification and quantification of individual snoRNAs was conducted with specific stem-loop reverse transcription primers, gene-specific forward primers and a universal reverse primer (Kramer, 2011). snoRNA expression levels were analyzed relative to 5.8S rRNA. Sequences of primers used for quantitative reverse transcription PCR (qRT-PCR) are provided in Table S6.

### RiboMeth-seq

5 μg total RNA was hydrolyzed in 50 mM bicarbonate buffer pH 9.2 and 10 mM MgCl_2_ at 95°C for 5 min, then put on ice. RNA cleavage was stopped by adding 2 μl of 0.5 M EDTA pH 8.0. The RNA was precipitated by adding 1/10 volume of 3 M sodium acetate and two volumes of 100% ice-cold ethanol, washed with 70% ice-cold ethanol and resuspended in nuclease-free water. RNA fragments were separated on a denaturing 6% acrylamide:bis-acrylamide (19:1), 8 M urea gel. The fragments in a size range of 20 to 40 nucleotides were extracted from the gel. The 5' ends of the RNA fragments were dephosphorylated using Shrimp Alkaline Phosphatase (rSAP; M0371S; New England Biolabs, Ipswich, MA, USA) as described in the manufacturer's protocol. The sequencing library was prepared using an NEB SmallRNA for Illumina kit (New England Biolabs, Ipswich, MA, USA) and sequenced as single-end reads with two technical replicates at the Deep Sequencing Core Facility (BioQuant) of Heidelberg University. The RNA sequences were trimmed and aligned to the human genome (GRCh38) using Geneious software (Auckland, New Zealand). The fraction of methylated rRNA and RMS scores were calculated as previously described (Birkedal et al., 2015).

### Three-dimensional visualization of differentially methylated sites

Known 2′*-O-*Me sites on human 18S and 28S rRNA (Taoka et al., 2018) were visualized in the human ribosome crystal structure with PDB accession 6QZP (Natchiar et al., 2017) using Pymol (v2.1.1). Our study and Taoka et al. (2018) use a 28S nucleotide numbering based on GenBank ID U13369 (nucleotides 7935 to 12,969), whereas the 28S sequence numbering in the PDB:6QZP crystal structure is based on NCBI Refseq NR_003287.4. Thus, we converted the 2′*-O-*Me sites into the NCBI Refseq NR_003287.4 numbering for visualization.

### Northern blotting

For northern blotting, 4 µg of total RNA was separated on a 1.2% glyoxal agarose gel or 8% polyacrylamide/urea gel. Isolated RNA was hydrolyzed and transferred to a Hybond+ nylon membrane (Roche Diagnostics, Mannheim, Germany) at 4°C using wet electro-transfer. The membrane was stained with Methylene Blue to confirm homogenous and efficient transfer. Radiolabeled probes (Table S5; Wise et al., 1983) were hybridized with the membrane in Church hybridization buffer (0.36 M Na_2_HPO_4_, 0.14 M NaH_2_PO_4_, 1 mM EDTA, 7% SDS) at 38–42°C overnight, and membranes were then washed and exposed to phosphor-imaging plates (Fujifilm, Tokyo, Japan). The imaging plates were scanned on an FLA-5100 scanner (Fujifilm, Tokyo, Japan), and quantification was performed using the AIDA software (Elysia-Raytest, Angleur, Belgium), in 1D-quantification mode. Some snoRNA expression levels were low and required 2–3 different probes per snoRNA and several days of exposure. The signal of each snoRNA was normalized to that of SNORD57 (SSU-A99). SNORD57, which has signal levels comparable to those of other snoRNAs, was chosen as control as neither the RMS score at its guided position nor the level of snoRNA expression changed significantly between P cells, Q cells and SIPS cells. Quantification relative to 5.8S rRNA gave similar results. Northern blotting was performed with HDF76 and HDF161 cells.

### Western blotting

Cells were lysed in RIPA buffer (150 mM NaCl, 0.5% sodium deoxycholate, 1% NP-40, 0.1% SDS, 50 mM Tris-HCl pH 8.0). After sonification with a Bioruptor Plus sonicator (Diagenode SA, Seraing, Belgium) for 30 cycles (30 s on/30 s off), SDS-PAGE sample buffer (60 µM Tris-HCl pH 6.8, 2% SDS, 10% glycerol, 0.0125% Bromophenol Blue and 1.25% β -mercaptoethanol) was added and mixed thoroughly. 4–15% Mini-PROTEAN TGX Gels (Bio-Rad Laboratories, Hercules, CA, USA) in Laemmli running buffer (25 mM Tris, 250 mM glycine and 0.1% SDS) were used for electrophoresis. The transfer to PVDF membranes (Bio-Rad Laboratories, Hercules, CA, USA) was performed at 25 V and 1.3 A for 3 min. Following blocking with 3% non-fat dry milk in PBS overnight, primary antibody incubation with anti-fibrillarin (ab4566; Abcam, Cambridge, UK) at a 1:2000 dilution or anti-β-actin (ab8224; Abcam, Cambridge, UK) at a 1:1000 dilution for 1 h, and secondary antibody incubation with IRDye 680RD donkey anti-mouse IgG (926-68072; LI-COR, Lincoln, NE, USA) at a 1:20,000 dilution for 1 h, the membrane was scanned on an Odyssey Infrared Imager (LI-COR, Lincoln, NE, USA). Each band was quantified using ImageJ (version 1.52 e; NIH, Bethesda, MD, USA) after brightness and contrast adjustments.

### Comparison of snoRNA and corresponding host gene expression

Previously published datasets for small RNA (GEO accession GSE95354; Terlecki-Zaniewicz et al., 2018) and mRNA expression (GEO accession GSE93535; Lämmermann et al., 2018) comparing quiescence with hydrogen peroxide-induced senescence were used for the analysis. Normalized count tables were imported into Qlucore Omics Explorer version 3.9.9 (Qlucore, Lund, Sweden) for statistical analysis and plotting. Differences between groups were determined by Tukey's range test following multi-factor ANOVA, adjusted for cell donors.

### Knockdown of snoRNAs using antisense oligonucleotides

ASOs were designed as previously described (Liang et al., 2011). ASOs used for targeting each snoRNA were five–ten–five RNA–DNA chimeric oligonucleotides linked with a phosphorothioate backbone (indicated by * in the sequences below), with ten deoxyribonucleotides flanked at both sides by five ribonucleotides modified with 2′*-O-*methylation (indicated by m in the sequences below). Modified ASOs targeting SNORD87 (mC*mA*mG*mC*mU*G*G*G*T*A*A*A*C*G*G*mC*mA*mA*mA*mA) and SNORD88A/B/C (mG*mA*mG*mC*mC*C*A*G*U*G*C*U*G*G*A*mC*mA*mU*mC*mA), as well as a scrambled control (mG*mA*mU*mA*mA*C*C*G*C*G*A*G*A*A*G*mA*mC*mC*mC*mU) were synthesized by Integrated DNA Technologies (Coralville, IA, USA). Sub-confluent cells (60–80%) were transfected with ASOs at a final concentration of 50 nM using either jetPRIME (Polyplus-transfection S.A, Illkirch-Graffenstaden, France) or Lipofectamine RNAiMAX (Life Technologies, Waltham, MA, USA), based on the manufacturer's instructions for reverse transfection. Cells were harvested at the indicated time points for further analysis.

### Proliferation assay

HDFs were seeded at 50,000 cells/well in six-well culture plates. The medium was not changed and cells were not passaged during the entire experiment. After plating, the cells were detached by trypsinization and counted using a Vi-CELL XR (Beckman Coulter, Brea, CA, USA) automated cell counter at the indicated time points.

### Cell cycle analysis

At 3 days after transfection, the cells were washed with PBS without calcium and magnesium and were harvested using trypsin. The cell pellet was resuspended in 2 ml F-12 medium containing 3 µg/ml of bisbenzimide Hoechst 33342 (Sigma-Aldrich, St. Louis, MO, USA) and incubated for 30 min at 37°C. After incubation, the samples were centrifuged at 170 ***g*** for 5 min, the supernatant was discarded, and the pellet was resuspended in 100 µl complete medium and measured using a Beckman Coulter CytoFLEX S flow cytometer (Beckman Coulter, Brea, CA, USA).

### Apoptosis assay by annexin V staining

The experiment was performed as previously described (Terlecki-Zaniewicz et al., 2018). Cells were stained 3 days after transfection with annexin V staining solution containing 200 ng/ml Pacific Blue-conjugated annexin V (#640918; Biolegend, San Diego, CA, USA), diluted in annexin V binding buffer (10 mM HEPES-NaOH pH 7.4, 140 mM NaCl, 5 mM CaCl_2_). Analysis was performed on a CytoFLEX S flow cytometer (Beckman Coulter, Brea, CA, USA). An excitation wavelength of 405 nm and a 450/45 band pass fluorescence channel were used to detect Pacific Blue-conjugated annexin V. Positive-control cells were treated with 1 µM staurosporin (S6942; Sigma-Aldrich, St. Louis, MO, USA) for 4 h. Flow cytometry data were analyzed with FloJo version 10.9.0 (Becton, Dickinson and Company, Franklin Lakes, NJ, USA).

### OPP assay to quantify global protein synthesis

Total protein synthesis was quantified as previously described (Nagelreiter et al., 2018). Cells were seeded at 3500 cells/cm^2^ density and incubated for at least 24 h at 37°C and 5% CO_2_. One sample was incubated with 50 µg/ml of cycloheximide for 15 min as a specificity control. After incubation, samples were treated with 25 µM OPP (*O*-propargyl-puromycin; NU-931-05; Jena Bioscience, Jena, Germany) for 30 min at 37°C, washed with PBS without calcium or magnesium, and harvested using trypsin. After centrifugation at 400 ***g*** for 5 min and discarding the supernatant, cells were fixed by adding 70% ethanol and subsequent incubation at 4°C overnight. Samples were then washed with PBS containing 115 mM Tris-HCl and 0.1% Triton X-100 and resuspended in PBS containing 115 mM Tris-HCl, 0.1% Triton X-100, 100 mM CuSO_4_ and 10 mM Alexa Fluor 488 azide (Life Technologies, Waltham, MA, USA). After 2 min of incubation, L-ascorbic acid was added to 2.5 mM. The samples were then incubated for 1 h at 37°C, washed with PBS containing 100 mM Tris-HCl, 2 mM MgCl_2_ and 1% Triton X-100 at pH 7.5, and then incubated in the same buffer containing 5 ng/ml DAPI for 15 min at room temperature. The cells were measured using a Beckman Coulter CytoFLEX S flow cytometer (Beckman Coulter, Brea, CA, USA) and subsequently analyzed with FlowJo 10.9 0 (Becton, Dickinson and Company, Franklin Lakes, NJ, USA). DAPI was used to exclude cell debris by gating.

### Statistical analysis

*t*SNE plots and the multi-group ANOVA statistical model with Tukey's post-hoc tests for analyzing the RMS data were generated in Qlucore Omics Explorer version 3.9.9 (Qlucore, Lund, Sweden). Prism 8 Version 8.4.3 (GraphPad Software, Boston, MA, USA) was used for all other statistical analyses and plotting. Specific details on the statistical tests and replicates are included in the figure legends. Sample sizes were defined based on the literature and the availability of biological material. No samples were excluded from the analysis.

## Supplementary Material



10.1242/joces.261930_sup1 Supplementary information
