# Supplementary material for: 2′-O-ribose methylation levels of ribosomal RNA distinguish different types of growth arrest in human dermal fibroblasts
Source: J Cell Sci. 2024 Feb 12;137(3):jcs261930. doi: 10.1242/jcs.261930 (PMC10911272; doi:10.1242/jcs.261930)
Supplement: Supplementary information [file joces-137-261930-s1.pdf]

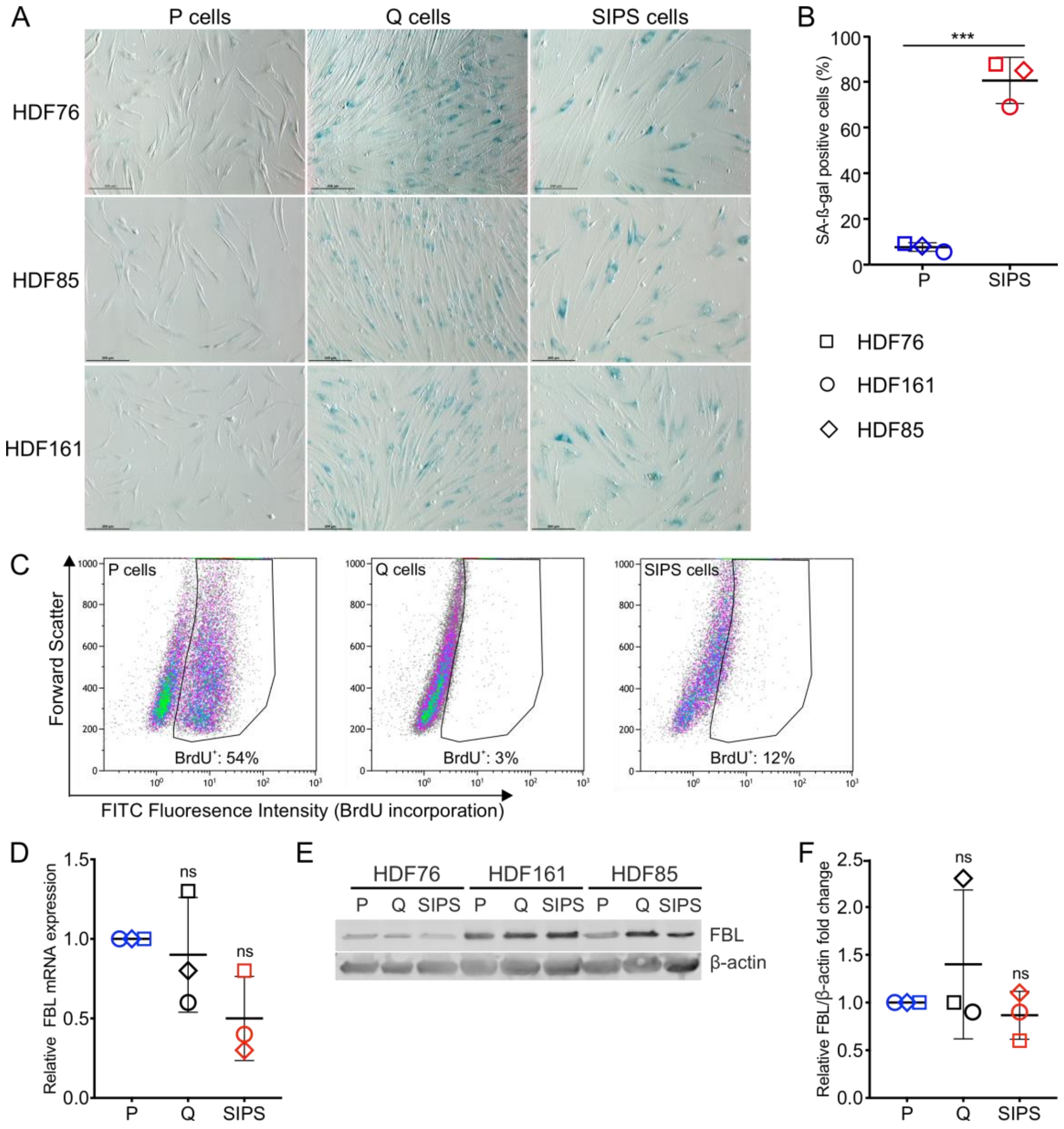

Fig. S1. Cellular senescence verification. Cellular senescence was confirmed by **(A-B)** SA-β-gal activity and **(C)** BrdU incorporation. **(A)** SA-β-gal staining for P, Q, and SIPS cells from three donors (HDF76, HDF85, HDF161) reveals the typical morphology of cellular senescence in SIPS cells. The scale bar represents 200 µm. **(B)** Quantification of SA-β-gal positive cells of P and SIPS cells. The error bars represent mean ± standard deviation. A two-tailed Student's t-test was used to determine the statistical significance. **(C)** BrdU incorporation measurement by flow cytometry for all three donors with a similar outcome. HDF76 is shown as a representative result. **(D)** Fibrillarin (FBL) mRNA expression via qRT-PCR (N = 3 biological replicates representing tissue donors). **(E)** Protein expression levels of FBL in P, Q, and SIPS cells by Western blot, β-actin was used as loading control (N = 3 biological replicates). **(F)** Relative fibrillarin/β-actin fold change by quantification of the Western blot from (E). One sample *t*-tests against an expected value of 1 were done for **(D)** and **(F)**, but did not reveal significant differences. α=0.05, ****P* < 0.001.

Journal of Cell Science • Supplementary information

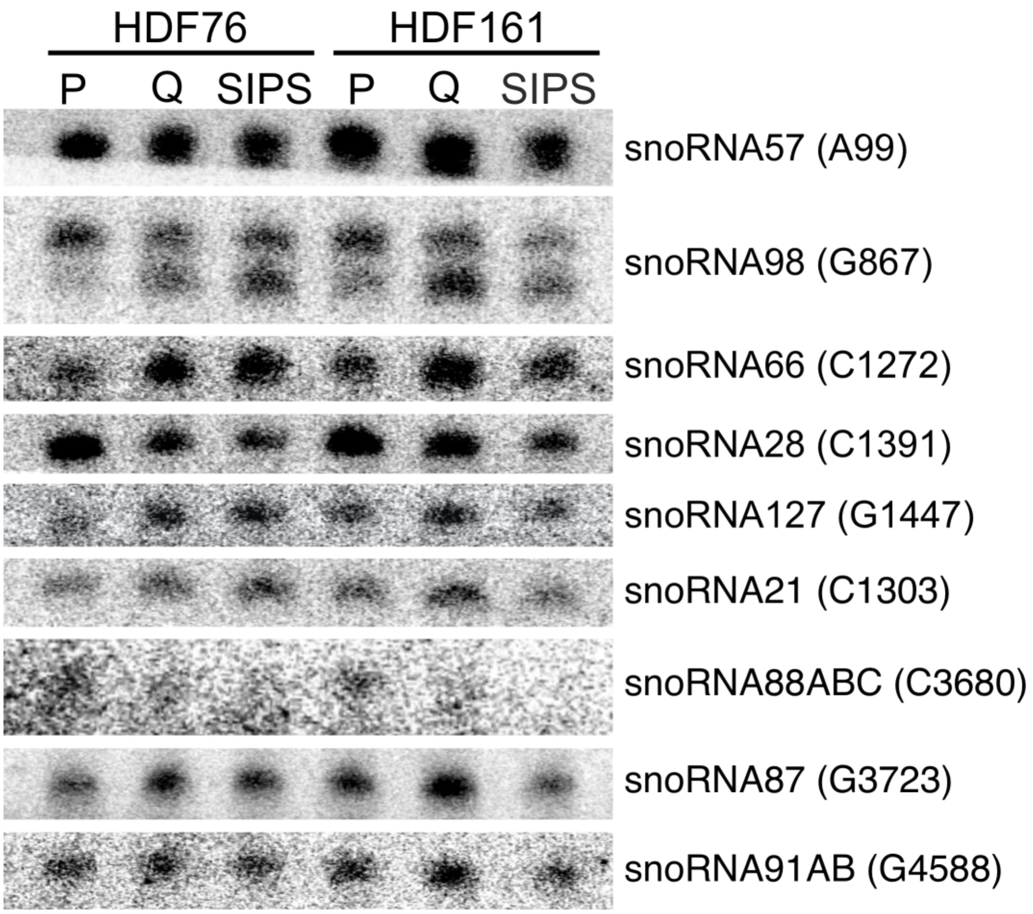

Fig. S2. Northern blot analysis of snoRNA expression. Total RNA was separated on a 1.2% agarose-glyoxal gel, transferred to a nylon membrane, and hybridized with probes complementary to snoRNAs. The predicted target site guided by the respective snoRNA is indicated in brackets. The quantification of the blots is shown in Fig. 2.

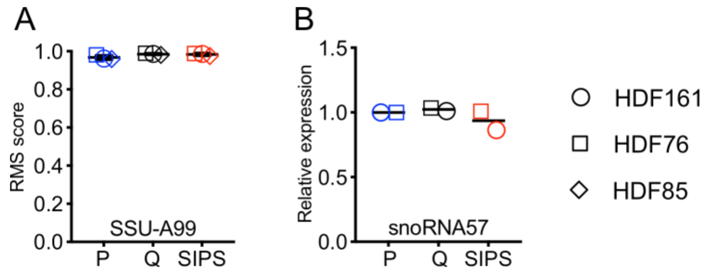

Fig. S3. Comparison of methylation and snoRNA expression for normalization control. **(A)** The RMS score at SSU-A99. **(B)** Quantification of SNORD57 levels from the northern blot, the expression level in P cells was normalized to 1.

Journal of Cell Science • Supplementary information

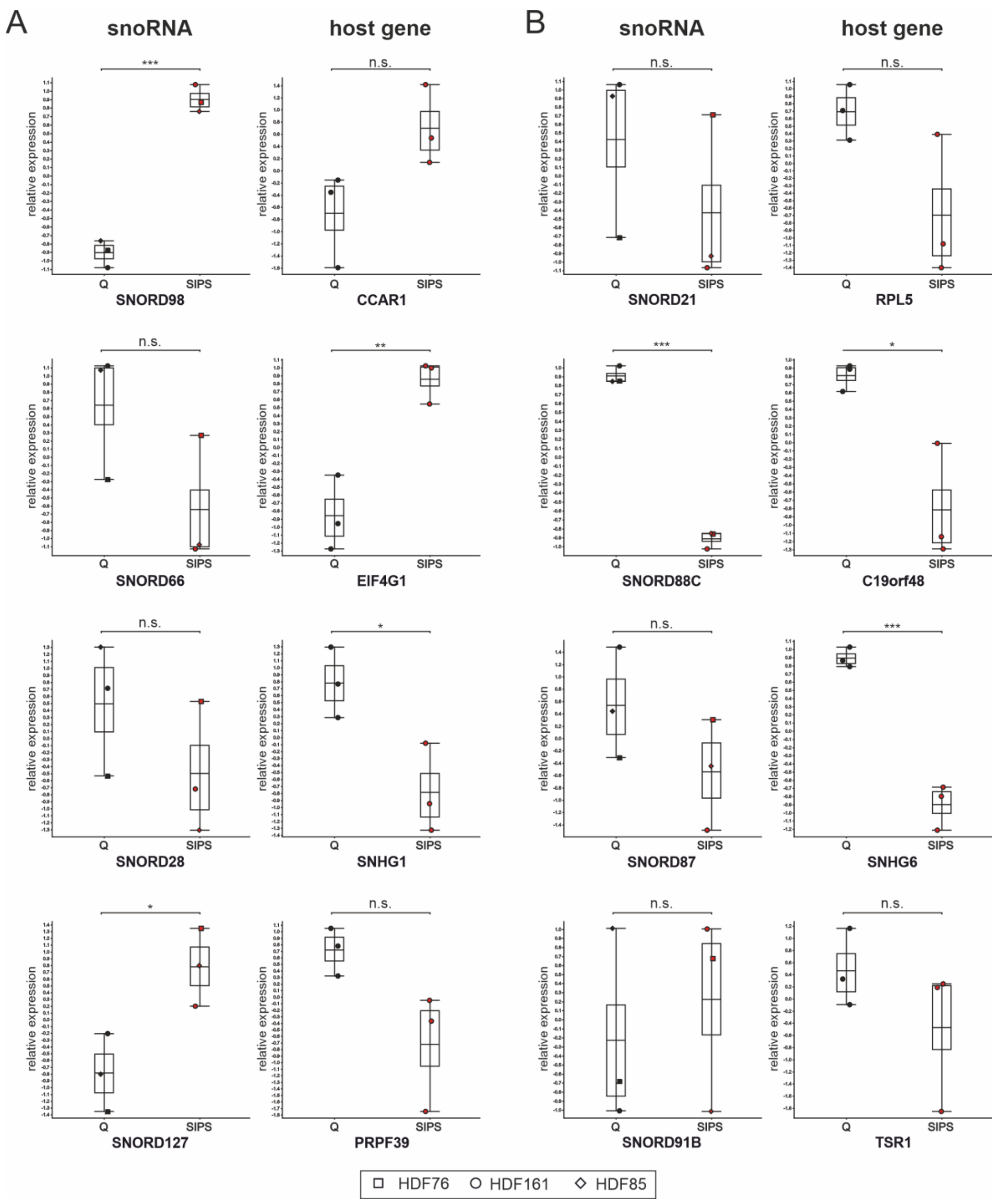

Fig. S4. Comparison of snoRNA and corresponding host gene expression. Expression of snoRNAs guiding methylation at the selected variable sites in 18S **(A)** and 28S **(B)** rRNA, as well as their corresponding host gene expression, are shown. Previously published datasets from our group for small RNA (GEO-accession: GSE95354) and mRNA expression (GSE93535) of quiescent vs. hydrogen peroxide-induced senescence were used for the analysis. N=3 biological replicates (=tissue donors) for snoRNAs, and N=3 independent experiments with HDF161 for host-gene expression. Error bars represent the range and boxes the 95% quartile. The statistical differences between groups were determined by the Tukey's range test following Multi-Factor ANOVA. α=0.05, *P < 0.05, **P < 0.01, ***P < 0.001.

Journal of Cell Science • Supplementary information

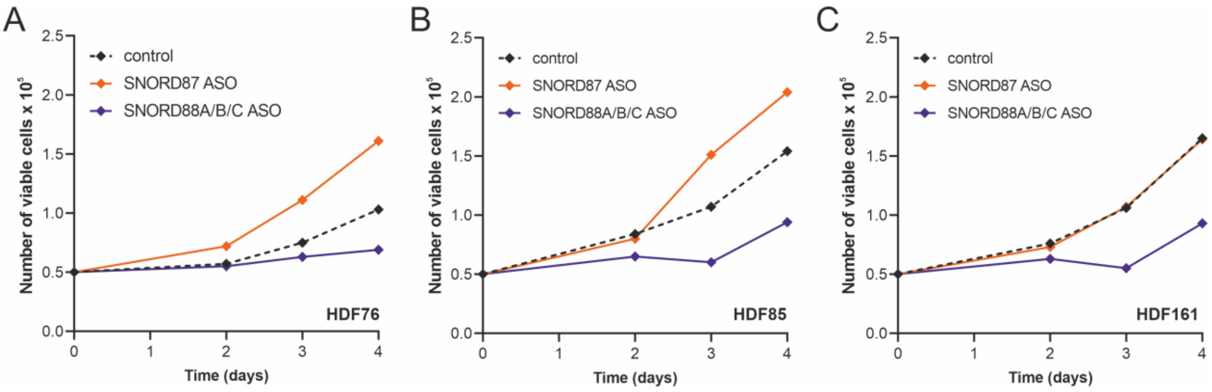

Fig. S5. SNORD87 and SNORD88A/B/C knockdown affect cell proliferation. HDF76 **(A)**, HDF85 **(B),** and HDF161 **(C)** were transfected with ASOs specifically targeting SNORD87 or SNORD88A/B/C, as well as a scrambled control ASO. Knockdown of SNORD87 promotes proliferation in HDF76 and HDF85, but not in HDF161. Knockdown of SNORD88A/B/C inhibits proliferation in all three cell strains compared to transfection with ASO control. N=1. An additional replicate of HDF76 is shown, which is not included in Fig. 4B.

Journal of Cell Science • Supplementary information

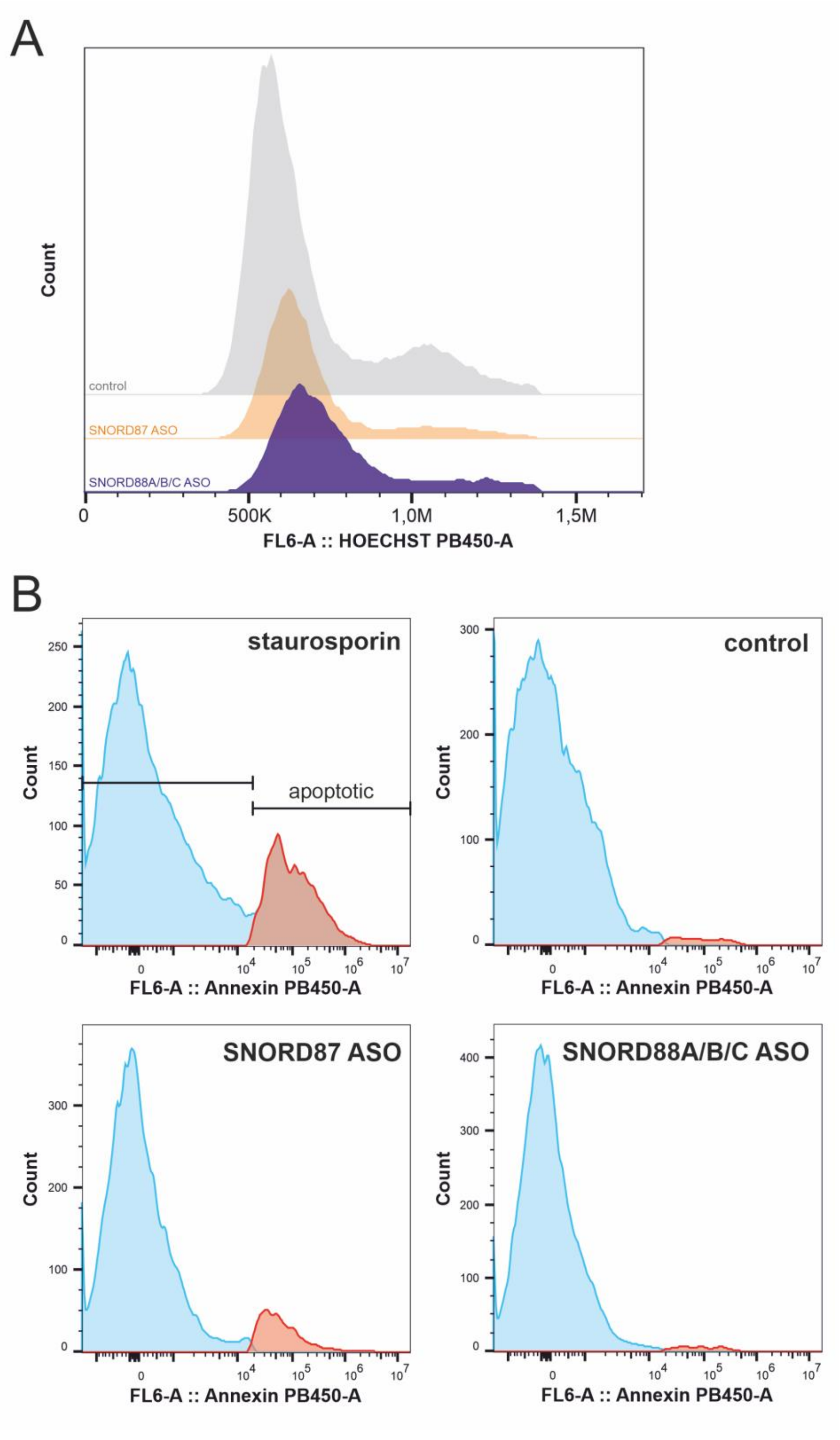

Fig. S6. Cell cycle and apoptosis analysis after SNORD87 and SNORD88A/B/C knockdown. HDF76 were transfected with ASOs specifically targeting SNORD87 or SNORD88A/B/C, as well as a scrambled control ASO. Histograms of representative replicates for the **(A)** cell cycle analysis using HOECHST33342 (Fig. 4C) and the **(B)** apoptosis assay using Annexin V-Pacific Blue (Fig. 4D) are shown. Histograms in **(A)** were vertically shifted for better visibility.

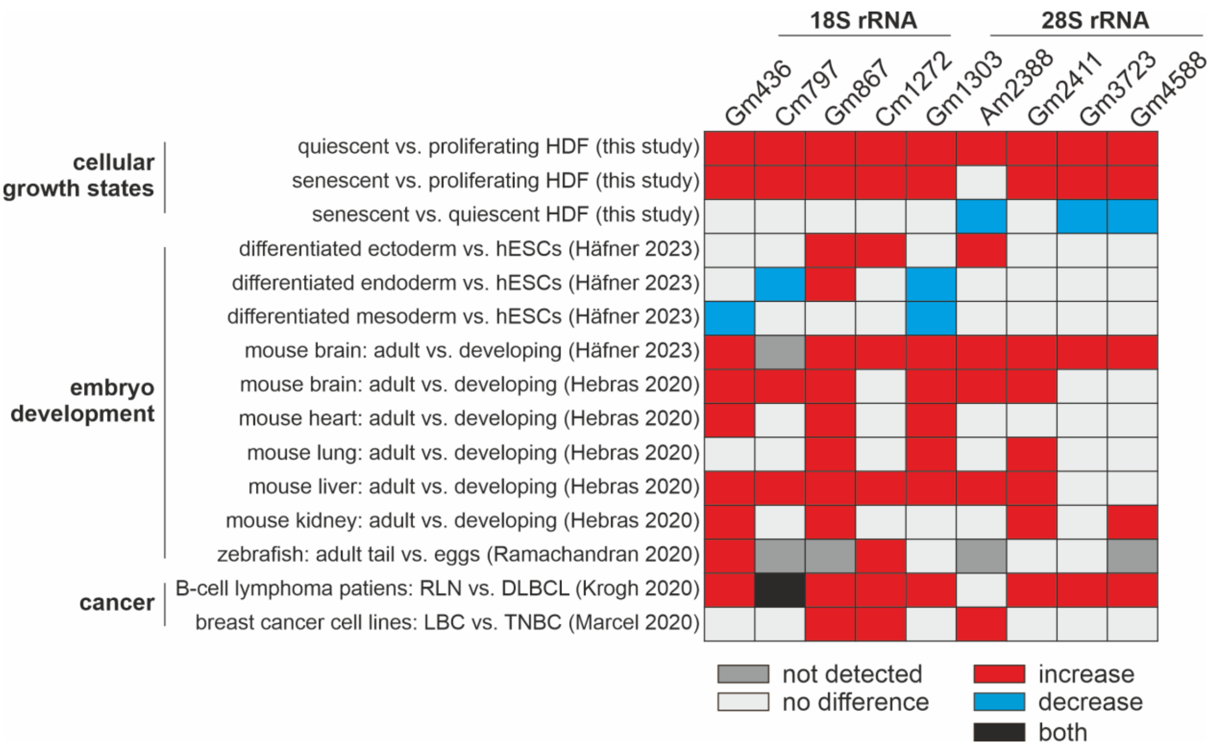

Fig. S7. Differentially modified variable sites are associated with embryo development and cancer progression in different tissue contexts. For all differentially modified 2-O-Me sites on 18S and 28S rRNA identified in the present study, there are differences in methylation levels between physiological states (proliferating, quiescent, and H_2O_2-induced senescence). Additionally, changes in modification levels throughout embryonic development and cancer reported in the literature are displayed. All references are included in the main reference list of the manuscript. "Both" refers to either an increase or decrease in individual DLBCL patients compared to the average methylation level in RLNs. Abbreviations: HDFs = human dermal fibroblasts, hESCs = human embryonic stem cells, DLBCL = diffuse large B-cell lymphoma, RLN = reactive lymph node, TNBC = triple-negative breast cancer, LBC = luminal breast cancer.

Journal of Cell Science • Supplementary information

Table S1. Characteristics of human dermal fibroblast strains from three donors

Name	Donor age (years)	Sex	Tissue origin	Max. PDs	Morphology of early-passage HDF	PD used for SIPS
HDF76	58	female	abdominoplasty	~55	papillary	20.5
HDF85	49	female	abdominoplasty	~35	papillary	13
HDF161	65	female	abdominoplasty	~40	reticular	15

Table S2. Differentially modified 2'-O-Me sites in 18S and 28S rRNA from three donors. Only sites showing differential regulation (Multi Group ANOVA, q < 0.1) are listed. P-values of Tukey's range test of pairwise comparisons are depicted.

Site	RNA	Multi Group ANOVA		Tukey's range test		
		p-value	q-value	proliferating/ quiescent	proliferating/ SIPS	quiescent/ SIPS
G436	18S	0.007	0.078	0.001	0.001	0.664
C797	18S	<0.001	0.012	<0.001	<0.001	0.092
G867	18S	<0.001	0.007	<0.001	<0.001	0.831
C1272	18S	0.004	0.069	0.001	<0.001	0.431
G1303	28S	0.006	0.074	0.001	0.001	0.692
A2388	28S	0.006	0.074	<0.001	0.146	0.002
G2411	28S	0.001	0.035	<0.001	<0.001	0.495
G3723	28S	0.002	0.042	<0.001	0.009	0.001
G4588	28S	0.001	0.035	<0.001	0.001	0.004

Journal of Cell Science • Supplementary information

Table S3. Average RMS scores at established 2’-O-Me sites in 18S rRNA from three donors. Sites showing differential regulation (Multi Group ANOVA, q < 0.1) are depicted in red. Sites showing the strongest plasticity and selected for further analysis in Fig. 2A are highlighted in italics.

	**Average of HDF76, 85, 161**			**St. dev. HDF76, 85, 161**		
**Established sites**	**P**	**Q**	**SIPS**	**P**	**Q**	**SIPS**
**A27**	0.987	0.988	0.989	0.011	0.010	0.009
**A99**	0.967	0.985	0.983	0.012	0.004	0.007
**U116**	0.953	0.971	0.957	0.013	0.003	0.019
**U121**	0.971	0.981	0.980	0.010	0.011	0.009
**A159**	0.990	0.995	0.995	0.005	0.002	0.002
**A166**	0.992	0.993	0.991	0.002	0.003	0.004
**U172**	0.965	0.977	0.972	0.018	0.012	0.015
**C174**	0.878	0.924	0.908	0.044	0.043	0.059
**U428**	0.922	0.966	0.962	0.049	0.014	0.022
**G436**	0.906	0.946	0.941	0.029	0.018	0.020
**C462**	0.932	0.940	0.953	0.044	0.026	0.018
**A468**	0.930	0.949	0.940	0.018	0.012	0.034
**A484**	0.969	0.972	0.969	0.008	0.016	0.015
**G509**	0.946	0.961	0.948	0.027	0.028	0.038
**A512**	0.859	0.919	0.857	0.071	0.044	0.094
**C517**	0.991	0.988	0.989	0.001	0.008	0.005
**A576**	0.979	0.992	0.984	0.010	0.003	0.009

Journal of Cell Science • Supplementary information

**A590**	0.973	0.989	0.988	0.013	0.000	0.003
**G601**	0.984	0.991	0.989	0.007	0.002	0.002
**U627**	0.955	0.951	0.947	0.017	0.031	0.009
**G644**	0.978	0.977	0.971	0.013	0.018	0.024
**A668**	0.974	0.973	0.974	0.005	0.010	0.010
**G683**	0.986	0.989	0.985	0.004	0.002	0.004
**C797**	0.847	0.937	0.925	0.027	0.015	0.015
**U799**	0.976	0.984	0.983	0.012	0.009	0.007
G867	*0.754*	*0.935*	*0.939*	*0.022*	*0.015*	*0.006*
**A1031**	0.974	0.982	0.984	0.021	0.014	0.011
C1272	*0.609*	*0.726*	*0.747*	*0.024*	*0.066*	*0.037*
**U1288**	0.958	0.961	0.962	0.028	0.036	0.033
**U1326**	0.969	0.971	0.964	0.005	0.014	0.010
**G1328**	0.960	0.965	0.963	0.014	0.012	0.007
**A1383**	0.977	0.979	0.932	0.009	0.016	0.059
C1391	*0.968*	*0.923*	*0.823*	*0.015*	*0.043*	*0.082*
**U1442**	0.946	0.981	0.970	0.039	0.009	0.019
G1447	*0.000*	*0.248*	*0.158*	*0.000*	*0.131*	*0.113*
**G1490**	0.999	0.999	0.999	0.000	0.000	0.000
**A1678**	0.944	0.956	0.958	0.020	0.031	0.022
**C1703**	0.982	0.985	0.979	0.008	0.005	0.008
**U1804**	0.945	0.971	0.957	0.016	0.005	0.014

Journal of Cell Science • Supplementary information

Table S4. Average RMS scores at established 2'-O-Me sites in 28S rRNA from three donors. Sites showing differential regulation (Multi Group ANOVA, q < 0.1) are depicted in red. Sites showing the strongest plasticity and selected for further analysis in Fig. 2B are highlighted in italics.

	**Average of HDF76, 85, 161**			**St. dev. HDF76, 85, 161**		
**Established sites**	**P**	**Q**	**SIPS**	**P**	**Q**	**SIPS**
**A389**	0.973	0.981	0.973	0.012	0.007	0.012
**A391**	0.947	0.973	0.971	0.007	0.007	0.008
G1303	*0.648*	*0.791*	*0.809*	*0.057*	*0.016*	*0.018*
**A1313**	0.948	0.933	0.937	0.023	0.048	0.042
**C1327**	0.743	0.780	0.687	0.079	0.017	0.022
**G1509**	0.926	0.925	0.912	0.027	0.032	0.025
**A1511**	0.973	0.975	0.967	0.015	0.013	0.025
**A1521**	0.995	0.995	0.995	0.003	0.004	0.003
**G1612**	0.992	0.993	0.990	0.006	0.004	0.007
**G1747**	0.964	0.984	0.985	0.028	0.012	0.009
**A1858**	0.996	0.996	0.995	0.001	0.002	0.003
**C1868**	0.783	0.828	0.796	0.088	0.086	0.072
**C2338**	0.949	0.948	0.939	0.008	0.011	0.014
**A2350**	0.990	0.989	0.986	0.005	0.007	0.011
**G2351**	0.981	0.971	0.956	0.006	0.015	0.026
**C2352**	0.930	0.927	0.910	0.016	0.026	0.038
**A2388**	0.912	0.943	0.920	0.032	0.021	0.027

Journal of Cell Science • Supplementary information

**U2402**	0.877	0.894	0.885	0.042	0.039	0.022
**C2409**	0.952	0.946	0.946	0.003	0.019	0.016
**G2411**	0.975	0.981	0.981	0.010	0.011	0.011
**A2774**	0.813	0.771	0.774	0.016	0.037	0.061
**C2791**	0.908	0.916	0.879	0.080	0.064	0.111
**A2802**	0.984	0.988	0.986	0.008	0.007	0.010
**C2811**	0.923	0.937	0.922	0.037	0.025	0.045
**U2824**	0.995	0.993	0.991	0.001	0.003	0.006
**C2848**	0.939	0.935	0.918	0.028	0.033	0.040
**G2863**	0.771	0.798	0.736	0.122	0.122	0.164
C3680	*0.831*	*0.757*	*0.552*	*0.032*	*0.105*	*0.200*
**A3697**	0.973	0.975	0.971	0.007	0.007	0.011
**A3703**	0.995	0.997	0.996	0.002	0.002	0.001
G3723	*0.560*	*0.734*	*0.631*	*0.132*	*0.110*	*0.149*
**A3739**	0.991	0.987	0.989	0.002	0.008	0.005
**A3764**	0.992	0.990	0.989	0.002	0.004	0.008
**G3771**	0.993	0.988	0.989	0.004	0.003	0.002
**C3787**	0.987	0.986	0.985	0.008	0.010	0.010
**U3797**	0.980	0.983	0.968	0.022	0.018	0.038
**A3804**	0.982	0.982	0.979	0.004	0.002	0.004
**A3809**	0.970	0.974	0.966	0.008	0.007	0.008
**C3820**	0.982	0.981	0.980	0.008	0.009	0.007

Journal of Cell Science • Supplementary information

**A3846**	0.917	0.910	0.914	0.011	0.008	0.023
**C3848**	0.880	0.902	0.918	0.072	0.065	0.040
**C3866**	0.948	0.959	0.945	0.011	0.006	0.014
**G3878**	0.980	0.978	0.980	0.006	0.011	0.008
**U3904**	0.972	0.967	0.973	0.016	0.024	0.015
**G3923**	0.864	0.865	0.860	0.083	0.105	0.102
**G4020**	0.839	0.876	0.871	0.094	0.098	0.090
**C4032**	0.971	0.965	0.967	0.012	0.020	0.021
**G4166**	0.983	0.985	0.981	0.004	0.001	0.002
**U4197**	0.997	0.996	0.997	0.001	0.001	0.000
**G4198**	0.976	0.962	0.965	0.009	0.023	0.020
**U4276**	0.987	0.986	0.984	0.003	0.003	0.003
**G4340**	0.977	0.984	0.980	0.005	0.007	0.004
**G4362**	0.959	0.963	0.963	0.017	0.009	0.005
**C4426**	0.965	0.972	0.963	0.005	0.003	0.006
**G4464**	0.994	0.996	0.996	0.003	0.003	0.001
**U4468**	0.974	0.974	0.977	0.013	0.023	0.013
**G4469**	0.973	0.972	0.971	0.009	0.013	0.008
**A4493**	0.993	0.994	0.993	0.004	0.005	0.003
**C4506**	0.996	0.996	0.996	0.000	0.000	0.001
**A4541**	0.956	0.977	0.982	0.018	0.009	0.005
**A4560**	0.940	0.962	0.964	0.020	0.014	0.016

Journal of Cell Science • Supplementary information

G4588	*0.638*	*0.779*	*0.719*	*0.063*	*0.042*	*0.043*
**U4590**	0.863	0.864	0.848	0.019	0.010	0.007
**G4593**	0.977	0.957	0.948	0.004	0.019	0.025
**G4607**	0.706	0.742	0.758	0.062	0.058	0.024

Table S5. Probes for Northern blots

**probe name**	**snoRNA/ pre-rRNA**	**sequence**
hSNORD91B(HBII-296B) _G4588_probe_rev	SNORD91A/B	AGTATCACACAGAAGTTGCATC
hSNORD91A(HBII-296A) _G4588_probe_rev		GAACCACACAGAGATTGCAT
hSNORD87(HBII-276) _G3723_probe_rev	SNORD87	AGCTGGGTAAACGGCA
hSNORD21 _G1303_probe_rev	SNORD21	TGCCATCAGTCCCGTC
hSNORD28(U28) _ C1391_probe_rev	SNORD28	TGCCATCAGAACTCTAACAT
hSNORD127_G1447_probe_rev	SNORD127	GTTTAGAGGGACTGTTGTCCT
hSNORD88C_C3680_probe_rev	SNORD88A/B/C	AGGTGTCAAAGGTCCTGG
hSNORD88B_C3680_probe_rev		AGTGCTGGACATCACGG
hSNORD88A_C3680_probe_rev		GTGCTGGACATCATGGAG
h5.8S-rRNA_ probe _rev	5.8S rRNA	GTGTCCTGCAATTCACATT
hSNORD66_C1272_probe_rev	SNORDD66	GTTCCATCATGGTGTCTCAG
hSNORD98_G867_probe_rev	SNORD98	GTTCCACACTGCATTTCAG
hSNORD57_A99_probe_rev	SNORD57	GTCAGGCTCAGACAGTTCAT
OMK1874	ITS2	GGCAAGAGGAGGGCGGA
OMK1873	ITS1	GTCCGGGCTCCGTTAATGATC

Journal of Cell Science • Supplementary information

Table S6. qRT-PCR primers

name	primer type	oligo Sequence
UniLoop	universal reverse primer	GTGCAGGGTCCGAGGT
snoR98	stem-loop_RT primer	GTTGGCTCTGGTGCAGGGTCCGAGGTATTCGCAC CAGAGCCAACGAGTTC
	forward primer	GGGGGAGTTATGATGTGTGTAAATC
snoR28	stem-loop_RT primer	GTTGGCTCTGGTGCAGGGTCCGAGGTATTCGCAC CAGAGCCAACTGCCAT
	forward primer	TGGGTCAGATGATTTGAATTGATAAG
snoR66	stem-loop_RT primer	GTTGGCTCTGGTGCAGGGTCCGAGGTATTCGCAC CAGAGCCAAC TTCCTC
	forward primer	GTGTGTTTCCTCTGATGACTTCC
snoR127	stem-loop_RT primer	GTTGGCTCTGGTGCAGGGTCCGAGGTATTCGCAC CAGAGCCAACTGGCAA
	forward primer	GTTGGCAACTGTGATGAAAGAT
snoR21	stem-loop_RT primer	GTTGGCTCTGGTGCAGGGTCCGAGGTATTCGCAC CAGAGCCAACGCTGCC
	forward primer	TGGGCTGAATGATGATATCCCA
snoR88C	stem-loop_RT primer	GTTGGCTCTGGTGCAGGGTCCGAGGTATTCGCAC CAGAGCCAACCTGGGG
	forward primer	GTTTCTGGGGCTCCCATGAT
snoR87	stem-loop_RT primer	GTTGGCTCTGGTGCAGGGTCCGAGGTATTCGCAC CAGAGCCAACTCTCAG
	forward primer	TGGGGACAATGATGACTTAAATTACTTTT

snoR91B	stem-loop_RT primer	GTTGGCTCTGGTGCAGGGTCCGAGGTATTCGCAC CAGAGCCAACAAAAGCC
	forward primer	TGGGGAAGAGCCAATGATGTTTTTAT
5S	forward primer	CATACCACCCTGAACGCG
	reverse primer	CTACAGCACCCGGTATTCCC
5.8S	forward primer	ACTCTTAGCGGTGGATCA
	reverse primer	ATCAATGTGTCCTGCAATTC
FBL	forward primer	ATGCTCGACACCCACACAAA
	reverse primer	CCTCCATTACGCAGGAAGGT
GAPDH	forward primer	CGACCACTTTGTCAAGCTCA
	reverse primer	TGTGAGGAGGGGAGA TTCAG
