# Supplementary material for: 2′-O-ribose methylation levels of ribosomal RNA distinguish different types of growth arrest in human dermal fibroblasts
Source: J Cell Sci. 2024 Feb 12;137(3):jcs261930. doi: 10.1242/jcs.261930 (PMC10911272; doi:10.1242/jcs.261930)
Supplement: Supplementary file 1 [file joces-137-261930_review_history.pdf]

2′-*O*-ribose methylation levels of ribosomal RNA distinguish different types of growth arrest in human dermal fibroblasts

Guohuan Yang, Maximilian Schmid-Siegel, Clemens Heissenberger, Isabelle C. Kos-Braun, Martina Prechtl, Gabriel Meca-Laguna, Marta Rocha, Anja Wagner-Schrittwieser, Vera Pils, Barbara Meixner, Koray Tav, Markus Hengstschläger, Johannes Grillari, Martin Koš and Markus Schosserer

DOI: 10.1242/jcs.261930

Editors: Maria Carmo-Fonseca and Michael Way

Review timeline

Original submission:	14 July 2021
Editorial decision:	17 August 2021
Resubmission:	27 December 2023
Accepted:	4 January 2024

Original submission

Original submission decision letter:

MS ID#: JOCES/2021/259137

MS TITLE: Cellular senescence and proliferation are associated with an altered ribosomal RNA methylation
AUTHORS: Guohuan Yang, Clemens Heisenberger, Isabelle Kos-Braun, Norbert Polacek, Johannes Grillari, Markus Schosserer, and Martin Kos
ARTICLE TYPE: Short Report

Dear Dr. Kos,

We have now reached a decision on the above manuscript.

To see the reviewers' reports and a copy of this decision letter, please go to: https://submit-jcs.biologists.org and click on the 'Manuscripts with Decisions' queue in the Author Area. (Corresponding author only has access to reviews.)

As you will see, the reviewers raise a number of substantial criticisms that prevent me from accepting the paper at this stage. They suggest, however, that a revised version might prove acceptable, if you can address their concerns. If you think that you can deal satisfactorily with the criticisms on revision, I would be pleased to see a revised manuscript. We would then return it to the reviewers.

We are aware that you may be experiencing disruption to the normal running of your lab that makes experimental revisions challenging. If it would be helpful, we encourage you to contact us to discuss your revision in greater detail. Please send us a point-by-point response indicating where you are able to address concerns raised (either experimentally or by changes to the text) and where you will not be able to do so within the normal timeframe of a revision. We will then provide further guidance. Please also note that we are happy to extend revision timeframes as necessary.

Please ensure that you clearly highlight all changes made in the revised manuscript. Please avoid using 'Tracked changes' in Word files as these are lost in PDF conversion.

© 2024. Published by The Company of Biologists under the terms of the Creative Commons Attribution License (https://creativecommons.org/licenses/by/4.0/).

I should be grateful if you would also provide a point-by-point response detailing how you have dealt with the points raised by the reviewers in the 'Response to Reviewers' box. Please attend to all of the reviewers' comments. If you do not agree with any of their criticisms or suggestions please explain clearly why this is so.

Journal of Cell Science is committed to maintaining the integrity of the scientific record. Please read our requirements for preparing your figures (jcs_revision.pdf, attached) to avoid a potential delay in the publication process or rejection on the basis of non-compliance with these guidelines. This guide also includes recommendations on improving figure layout to help reviewers and readers appreciate your data.

At this stage, we also ask you to ensure your manuscript complies with our formatting guidelines – please see here for details. Provided you are able to fully address the referees' comments, we are positive about publication of your paper (we accept over 95% of revision submissions) and therefore hope you won't mind any extra work involved in reformatting your manuscript at this point.

**Please note that, as per our policy, HIGH-CONTRAST GELS AND BLOTS ARE UNACCEPTABLE (i.e. no white backgrounds). If you supply gels/blots with white backgrounds, we will ask you for the original data for verification, which will delay the processing of your manuscript.

Competing interests
Please note that if any author included on the manuscript has financial, personal or professional associations that could be perceived as interfering with the objectivity of their scientific judgement (for examples, see here), they must provide a disclosure statement, which will be published at the end of article. It is the responsibility of the corresponding author to ensure that all competing interests have been disclosed.

To submit your revised manuscript, please go to:
https://submit-jcs.biologists.org and click on the 'Submit a revision' link within the Author Area.

When preparing your revised manuscript, please bear in mind the following:

The length limit for Research Articles is 8000 words, and 3000 for Short Reports. This word count does not include the title page, abstract, materials & methods, or reference list but does include figure legends.

Please note that the original source files for text, tables and all figures will be required. Do not upload a single pdf containing tables and figures but instead provide a separate file for each item (i.e. a Word file containing the main text, tables and legends, and a separate file for each figure - see here for required file formats).

Supplementary material may include movies, figures and tables. Please collate supplementary figures and tables into a single PDF file. If your tables are very large, or you wish readers to be able to export and/or manipulate the data, we would prefer you to submit them as Microsoft Excel files. Movies and cover image suggestions can be uploaded separately.

Supplementary guidelines
Please see our website for further guidelines on supplementary material.

Section Order
Article sections should appear in the following order: Introduction, Results, Discussion, Materials and methods.
Please note that revised manuscripts will be returned if they do not follow the specified order.

Submission checklist
We are now requesting that authors fill in and upload a submission checklist with their manuscript. This form asks authors to confirm that they have followed best practice guidelines regarding experimental subjects, data reporting and statistics. The checklist is based on the NIH Principles

© 2024. Published by The Company of Biologists under the terms of the Creative Commons Attribution License (https://creativecommons.org/licenses/by/4.0/).

and Guidelines for Reporting Preclinical Research and is intended to help ensure high standards for reporting and to aid reproducibility.

Please fill in and upload the submission checklist into the supplemental files area. You can find a copy of the checklist here.

Funding info missing
To help you meet your funder requirements to report all financial support received, we require authors to provide details of all their funding sources during submission.

To add these details, go to: http://submit-jcs.biologists.org, enter your 'Author Area' and click on the queue 'Papers Returned for Your Attention'.

Click on the section ‘Funding Information’, where you will see instructions on how to enter your funder names and grant numbers.

The funding data submitted should match the information provided in the paper's Funding section. Please provide funder names in full.

It is important that this information is accurate as your funding data will be deposited with Crossref, and used by funder agencies to monitor the outputs of their support.

In order to promote timely publication, we generally ask that the revision be completed within 90 days from the date of this letter. However, as noted above, we recognise that this may not be possible under the current circumstances and will be happy to grant an extension where this is needed: please just contact the Editorial Office.

*** Please DO NOT tick the "Resubmission" box if you are re-uploading files. This is only intended for manuscripts which have either been rejected following the peer review process, or which have exceeded the revision submission deadline.***

I look forward to receiving your revised manuscript.

Reviewer 1 Advance Summary and Potential Significance to Field:
Using the method of RiboMeth-seq to measure 2´O-Me on rRNA the authors show novel data on the status of this modification in human proliferating fibroblasts, during quiescence and during premature stress induced senescence. The authors also show data on rRNA levels and processing according to Northern blot in these cells and also measure snoRNA and fibrillarin. The paper, in the shape or a short report, reveals that there are changes in these modifications and that modulation of certain snoRNA can affect proliferation, marginally, of fibroblasts and some cancer cell line.

Reviewer 1 Comments for the Author:
The manuscript contains data on the methylation of rRNA in ribosome biogenesis process, rRNA status in HDFs and some snoRNA expression and knockdown data.

Paper is well written, results clear and nicely presented. The paper has high technical level and experiments are not trivial.
The data is interesting and the authors make some points that it differs from several studies. But, maybe it is not so different from previous studies in the field as the authors give the impression of in the text.
For example, I think the authors need to more carefully consider the various senescence inducing conditions in their study versus those of Lessard et al and Nishimura et al. Depending on the use of oncogenes (Ras, Myc) or looking strictly at replicative senescence, or if using mouse fibroblasts or human fibroblasts some distinctions can be noted both in amount of new rRNA made and processing. I think this can be addressed a bit more carefully, rather than implicating methodological problems (although it is not spelled out in text, this is the impression at least I as a reader got), it could as well be meaningful biological differences between the cell culture models.

A more careful approach would also have included uridine-labeling and proper pulse chase analysis of rRNA.

© 2024. Published by The Company of Biologists under the terms of the Creative Commons Attribution License (https://creativecommons.org/licenses/by/4.0/).

The same reasoning may apply to the methylation measurements. While there has been fierce discussions on the method around some details, there may exist biologically significant differences. Moreover, fibrillarin may play a more important role in cancer cells than in primary fibroblasts (just as an example). I think the authors can address this, and similar issues, in a slightly better and more informed way.

The manuscript, although a short report, is not very well aligned, and data is mostly correlative in nature and one cannot really deduce effects from snoRNA knockdown rRNA state and to a biological endpoint in a good way. The abstract suggest that depletion of multiple individual snoRNAs impact on proliferation in a positive way, while this is not really the case from the results more like one positive data point on one snoRNAs than a general phenomenon. There is also a mix-in of data from cancer cell lines (HeLa) and this is a bit confusing for the reader since the paper is so focused from start on the fibroblast senescence.

I think there are quite a few limitations in this study but the main issue is that it is difficult to connect the various part of the study to create a meaningful biological endpoint / result rather than correlative measurements of modifications of rRNA to some snoRNA function, to proliferation (cell number). There is not even a measurement of total RNA or protein synthesis to complement with.

*****

Reviewer 2 Advance Summary and Potential Significance to Field:
Emerging evidences suggest that RNA modifications changes might contribute to alter RNA function and regulation.
In this study, Yang et al were analysing changes in ribosomal RNA 2´O methylation in quiescent, senescent and proliferating cells. The authors conclude that cellular senescence and proliferation are associated with specific alteration of rRNA 2´O methylation.
Overall, the study is of potential interest and looks technically well performed, however I have a few concerns regarding the strength of the proposed conclusions.

Reviewer 2 Comments for the Author:
Specific comments:
1)Changes in rRNA 2´O Methylation:
a) In total, the variations are rather limited to few positions and the observed variations are in many cases of modest magnitude. Biological variations can also be well seen in the rRNA processing analysis (Figure 2), suggesting that making conclusions of 3 donors' samples might be taken with a greater care considering that many secondary aspects are not fully controllable/known to the authors. I believe the authors should better highlight and discuss some of the possible drawbacks of the current study (some of the results seem to be dependent on cellular origin too, see below).
b) The authors do not really discuss whether there is any visible pattern when it comes to the 2D/3D positioning of the altered rRNA
modifications and importantly how these changes may directly influence ribosome function.
c) The title is a bit misleading in the sense that it almost implicitly reads somewhat like large changes of methylation at multiple positions is occurring. Moreover, the title should be more precise and less general, as well, since the analysis covers 2´O methylations, and not the other known types of rRNA methylations, and to integrate (as mentioned above) the paucity of the observed alterations should be made clearer.

2) Effect of rRNA methylation on cellular proliferation:
a) Are there any reasons to show only the HDF76 SNORD87/88 knock-down in Figure 3 and the average of the three HDF knockdown in supplementary
Figure 4? It would be critical to show how the HDF samples behave individually considering the underlying putative biological heterogeneity.
b) SNORD87 knockdown has no effect on proliferation in NIH3T3 or HeLa cells which is somehow in contradiction with the conclusions of the authors.
The authors should comment on that.
c) Based on the author´s conclusions, one could expect that SNORD87 knockdown should delay entrance into senescence or alternatively increasing SNORD87 (and the resulting 2´O methylation) may have a positive effect on cell proliferation?

© 2024. Published by The Company of Biologists under the terms of the Creative Commons Attribution License (https://creativecommons.org/licenses/by/4.0/).

Same should be equally true for SNORD127? It is also not fully clear why the authors only focus on Hela cells in this case (which was unaffected with SNORD87). What about NIH3T3 and/or the HDF donor cells?
These types of experiments would strongly strengthen the proposed direct effect of these rRNA modifications alteration on the regulation of cell proliferation.

*****

Reviewer 3 Advance Summary and Potential Significance to Field:
The study aims to better understand the potential role of rRNA 2'-O-ribose methylations, notably during cell senescence. It is part of an emerging field of research that aims to describe the extent to which ribosomes can be heterogeneous. Using RiboMeth-seq, the authors profiled 2'-O-methylations in rRNA of proliferating vs senescent vs quiescent human cells. This made it possible to reveal changes in the methylation levels at a very few sites rRNA sites (n=9). The authors also noticed some alterations in pre-rRNA processing. Of note, methylation levels at some differentially-methylated rRNA sites correlate with expression of the cognate SNORD. Finally, SNORD loss-of-function experiments are accompanied by altered cell proliferation. To my knowledge, this is one of the first, if not the first, study of rRNA methylation profiles in senescent cells. This type of study suggests that changes in rRNA methylation are dependent on the quiescence/senescence/proliferation state.

Reviewer 3 Comments for the Author:
I find this short manuscript interesting but before considering its publication, I would like the authors to clear up some ambiguities. In my view, some statements are over-interpreted and deserve to be nuanced in order to be more in line with the observed results. Addressing #3 and #8 seems to me to be a priority. I am not an English speaker and I would not allow myself to make relevant comments.

#1 - In my view, the title is somewhat ambiguous. Based on such a title and the abstract, one could easily interpreter that the authors have identified rRNA sites whose methylation changes are specifically associated with the senescence state. As I understand it, the situation is more complex. As stated by the authors: "The SSU-C1391, G1447, and LSU-C3680, G3723 methylation in SIPS differed from P or Q cells suggesting a specific association with SIPS, however the biological variance between donors at these sites precludes a conclusion from the current data". I therefore invite the authors to qualify their statement. If, on the other hand, the authors believe that they have truly identified senescence-specific rRNA methylations, then these latter should be highlighted more explicitly and discussed accordingly.

#2 - "In summary, 2'-O-Me profiling revealed a distinct pattern between senescent, quiescent and proliferating cells, consistent with the variable sites identified recently in cancer cells (Marcel et al., 2020)". It would be interesting if the authors would give more information on these differentially-methylated rRNA sites. Are they sites known to be variable in other biological systems? Are they altered in cancers? It seems to me that it would also be reasonable for the authors to state clearly that the observed changes are relatively minor both in terms of the number of sites and the magnitude of the changes. I also get the impression that the changes are mostly in rRNA sites that are never 95-100% methylated in any of the cell types analyzed. This should also be mentioned and discussed accordingly. Such a comparison could be given in the supplementary data.

#3 - "Even small methylation changes were accompanied by comparable alterations in the snoRNAs levels (Figure 1b, d; 61 Supplementary Figure 3), suggesting that the observed hypomethylation might be due to the limiting levels of the snoRNAs". As I understand it, the measurements of SNORD expression by Northern are not consistent with those made by RT-qPCR (Figure 1d), especially when comparing proliferating vs quiescent cells. Could the authors provide explanations for such discrepancies? What about the expression levels of SNORD host-genes transcripts? In my view, this could be easily addressed.

#4 - "These results indicate that the changes in methylation and snoRNA expression between P and SIPS/Q cells are not just a side-effect but are more intrinsically linked to cell proliferation". Because the authors did not measure rRNA methylation levels in SNORD-depleted cells, this conclusion is, in my view, over-interpreted and should be modified accordingly.

© 2024. Published by The Company of Biologists under the terms of the Creative Commons Attribution License (https://creativecommons.org/licenses/by/4.0/).

#5 - Figure 3_ panel a - As I understand it, control cells have also been transfected with a non-targeting oligo. If this is the case, then it should be stated more explicitly in the figure and its legend.

#6 - Figure 3_ panel b - I am not sure I understand the rationale for mutating SNORD127 in HeLa cells. Indeed the corresponding site (SSU-G1147) is methylated up to 70% in HeLa cells (doi: 10.1093/nar/gkw482) whereas the authors find it largely under-methylated, not to say non-methylated in this study. In this regard, how sure are the authors that a RMS score around 0-30 is not simply technical background noise?

#7 - “A cut between the T and A three nucleotides upstream of the D-box motif (CUGA) was inserted in SNORD127 (Supplementary Figure 5a,b)”. As I understand it, the authors only modified the antisense element of SNORD127 via CRISPR-Cas9. If this is the case, I do not understand why the expression level of SNORD127 is reduced. This is an unexpected result since only the 5’-3’ terminal stem structure is required for efficient processing and accumulation. Can the authors comment? I also invite the authors to draw the WT and mutated antisense segments of SNORD127 in Figure 3. This will help the reader.

#8 – “Two days after transduction, mKate2- positive cells were isolated by FACS. After antibiotic selection with blasticidin and puromycin for two weeks, dilution cloning was performed in 96-well plates, single clones were picked, and analyzed by Sanger-sequencing (Eurofins)”. How many independent and randomly-picked SNORD127M clones did the authors analyse when they measured cell growth (Figure 3 panels b,d) ? Moreover, were the control cells also transduced with irrelevant constructs and underwent the same dilution cloning procedure? In my view, the use of matched control clones is essential to avoid bias and other artefacts. This is all the more important as the analysis of mutated Hela cells is very minimalist. Indeed, one would have expected such studies to be accompanied by analyses of cell cycle and apoptosis. Finally, and as stated above, the authors did not profile rRNA methylation in SNORD127M HeLa cells. Perhaps primer extension could be made with low dNTPs concentration.

Resubmission

Dear Dr. Carmo-Fonseca,

We are delighted that the reviewers think the manuscript is timely and interesting to the readership of the Journal of Cell Science. We thank the reviewers for their constructive criticisms, which we feel have significantly increased the quality of our manuscript. Our replies to their concerns are below.

Reviewer 1

Using the method of RiboMeth-seq to measure 2'O-Me on rRNA the authors show novel data on the status of this modification in human proliferating fibroblasts, during quiescence and during premature stress induced senescence. The authors also show data on rRNA levels and processing according to Northern blot in these cells and also measure snoRNA and fibrillarin. The paper, in the shape or a short report, reveals that there are changes in these modifications and that modulation of certain snoRNA can affect proliferation, marginally, of fibroblasts and some cancer cell line.

The manuscript contains data on the methylation of rRNA in ribosome biogenesis process, rRNA status in HDFs and some snoRNA expression and knockdown data.

Paper is well written, results clear and nicely presented. The paper has high technical level and experiments are not trivial.

Thank you very much for your accurate summary and for appreciating our manuscript's writing and presentation and our study's technical level.

© 2024. Published by The Company of Biologists under the terms of the Creative Commons Attribution License (https://creativecommons.org/licenses/by/4.0/).

The data is interesting and the authors make some points that it differs from several studies. But, maybe it is not so different from previous studies in the field as the authors give the impression of in the text.

For example, I think the authors need to more carefully consider the various senescence inducing conditions in their study versus those of Lessard et al and Nishimura et al. Depending on the use of oncogenes (Ras, Myc) or looking strictly at replicative senescence, or if using mouse fibroblasts or human fibroblasts some distinctions can be noted both in amount of new rRNA made and processing. I think this can be addressed a bit more carefully, rather than implicating methodological problems (although it is not spelled out in text, this is the impression at least I as a reader got), it could as well be meaningful biological differences between the cell culture models.

We appreciate this point and agree that cellular senescence is a highly heterogeneous cell phenotype and that the individual characteristics are strongly related to the senescence inducer (replicative, oncogenes, oxidative stress) and the cell type (species and tissue). We apologize for not being sufficiently clear about the different types of cellular senescence in the original version of the manuscript. To address this issue, we expanded the following paragraph in the introduction section (lines 60-68):

Ribosomes represent a promising target for senotherapies, as delayed ribosomal RNA (rRNA) processing promotes induction of senescence by activation of p53 in response to oncogenic and replicative stress (Nishimura et al., 2015), and pre-ribosome accumulation maintains the senescent state by engaging pRb in models of oncogene-induced and replicative senescence (Lessard et al., 2018). Importantly, **cellular senescence is a highly heterogeneous phenotype strongly influenced by the senescence inducer and cell type**, among other factors. Recently, Papaspyropoulos and colleagues demonstrated that replication-, genotoxic stress-, and oncogene-induced senescence commonly reduce translational efficiency, while particular changes in translational regulation depend on the specific senescence inducer (Papaspyropoulos et al., 2023).

For the same reason, we specified the senescence inducer in the header of the description of the ribosome biogenesis data (line 137):

Hydrogen peroxide-induced senescence alters rRNA processing

Moreover, we added the following paragraph to the discussion (lines 208-211):

Similarly, we concentrated on HDF and one inducer of cellular senescence and quiescence each. Further studies will be required to determine if the discovered mechanisms are universal and if the same sites are differentially modified in other cell types, in oncogene-induced or replicative senescence, and in serum-starvation-induced quiescence.

A more careful approach would also have included uridine-labeling and proper pulse chase analysis of rRNA.

We thank the reviewer for this suggestion. We have already acquired evidence that RNA and global protein synthesis, quantified by flow cytometry after 5-ethynyluridine (EU) and O-propargyl-puromycin (OPP) labeling, increase in our senescence model. However, our current manuscript's primary focus is identifying rRNA modifications related to cellular senescence and quiescence. Thus, we would like to refrain from including the results of the EU-labeling experiment here. Instead, we plan to publish these data in a separate manuscript on elevated protein synthesis in senescent cells and the effects of senotherapies to mitigate this increase.

The same reasoning may apply to the methylation measurements. While there has been fierce discussions on the method around some details, there may exist biologically significant differences. Moreover, fibrillarin may play a more important role in cancer cells than in primary fibroblasts (just as an example). I think the authors can address this, and similar issues, in a slightly better and more informed way.

We fully agree with the reviewer and are sorry that the first version of the manuscript was

© 2024. Published by The Company of Biologists under the terms of the Creative Commons Attribution License (https://creativecommons.org/licenses/by/4.0/).

probably too focused on details and consequently failed to put our data in a broader biological context. We have restructured, expanded, and rewrote several passages to address this issue. The new Figure 1E shows the 2'-O-Me modifications in their structural context, and Figure S7 compares the identified sites with the literature, establishing a link between these sites, development, and cancer. We also added Figures 1A and B to convey that rRNA modification signatures can distinguish the different cellular states, even if changes at individual sites are subtle. Similarly, to remain focused on human fibroblasts, we removed all data on cancer cell lines as their physiology significantly differs from primary cells.

Also, we agree that fibrillarin might limit the deposition of specific modifications in the context of cancer cells, which require strong upregulation of total protein synthesis to maintain their elevated growth rate. At the same time, this appears to differ in our primary fibroblasts, where snoRNAs seem to represent the rate-limiting entity. We aimed to clarify this by modifying the following passage (line 126- 135):

In a recent study, the expression levels of FBL affected methylation at specific positions in the HCT116 human colon carcinoma cell line (Sharma et al., 2017). However, to the contrary, in our primary fibroblast model, the methylation in Q/SIPS and P cells did not correlate with FBL expression, which was decreased in SIPS cells from all donors (Fig. S2). Instead, methylation at eight selected hypomodified sites, four on the SSU and four on the LSU, correlated with the expression of snoRNAs guiding their modification. Even small methylation changes were accompanied by comparable alterations in the snoRNA levels (Fig. 2A,B, S3, S4). The snoRNA expression correlated in some, but not in all cases, with the respective host gene (Fig. S5). Notably, levels of all snoRNAs for the hypomodified sites were low, suggesting that the observed hypomethylation might be due to the limiting amounts of the snoRNAs.

The manuscript, although a short report, is not very well aligned, and data is mostly correlative in nature and one cannot really deduce effects from snoRNA knockdown rRNA state and to a biological endpoint in a good way. The abstract suggest that depletion of multiple individual snoRNAs impact on proliferation in a positive way, while this is not really the case from the results more like one positive data point on one snoRNAs than a general phenomenon. There is also a mix-in of data from cancer cell lines (HeLa) and this is a bit confusing for the reader since the paper is so focused from start on the fibroblast senescence.

We thank the reviewer for the comment and are sorry that this critical point was not made clear enough in the first version of the manuscript. Accordingly, we modified the abstract as follows to avoid overstatements (line 36-39):

A knockdown of SNORD87, guiding a hypermethylated position in non-proliferating cells, was sufficient to boost cell proliferation. Conversely, depletion of SNORD88C guiding a hypomethylated site caused decreased proliferation without affecting global protein synthesis or apoptosis.

As mentioned above, we followed the reviewer's suggestion to avoid mixing cancer and primary cells by removing all cancer cell data and focusing the manuscript entirely on HDF.

I think there are quite a few limitations in this study but the main issue is that it is difficult to connect the various part of the study to create a meaningful biological endpoint / result rather than correlative measurements of modifications of rRNA to some snoRNA function, to proliferation (cell number). There is not even a measurement of total RNA or protein synthesis to complement with.

In response to the reviewer's concern, we now included data on cell cycle progression (Fig. 5C), apoptosis (Fig. 5D), and total protein synthesis (Fig. 5E) after snoRNA depletion and carefully restructured the whole manuscript. Thereby, we hope to connect our findings better and provide a more streamlined narrative. Still, we would like to emphasize that our short report's scope is to (1) provide the first evidence that rRNA modification patterns can distinguish different cellular growth conditions and (2) present proof of principle that specific growth-associated rRNA modifications can be targeted via snoRNA depletion to modulate the growth behavior of primary cells. A detailed characterization of specific sites and underlying mechanisms will be the subject

© 2024. Published by The Company of Biologists under the terms of the Creative Commons Attribution License (https://creativecommons.org/licenses/by/4.0/).

of further studies.

Reviewer 2

Emerging evidences suggest that RNA modifications changes might contribute to alter RNA function and regulation. In this study, Yang et al were analysing changes in ribosomal RNA 2'O methylation in quiescent, senescent and proliferating cells. The authors conclude that cellular senescence and proliferation are associated with specific alteration of rRNA 2'O methylation.

Overall, the study is of potential interest and looks technically well performed, however I have a few concerns regarding the strength of the proposed conclusions.

Thank you very much for considering our study interesting and technically sound.

Specific comments:

1) Changes in rRNA 2'O Methylation:

a) In total, the variations are rather limited to few positions and the observed variations are in many cases of modest magnitude. Biological variations can also be well seen in the rRNA processing analysis (Figure 2), suggesting that making conclusions of 3 donors' samples might be taken with a greater care considering that many secondary aspects are not fully controllable/known to the authors. I believe the authors should better highlight and discuss some of the possible drawbacks of the current study (some of the results seem to be dependent on cellular origin too, see below).

We fully agree with the reviewer and are sorry we were not sufficiently explicit about this point in the first version of our manuscript. We refined the statistical analysis of the RiboMeth-seq data and now provide tSNE plots (Fig. 1A, B), indicating the large variability introduced by the tissue donors and the possibility of adjusting the statistical model for them. Thereby, we identified nine differentially modified sites on both ribosomal subunits. Additionally, we added the following paragraph to the discussion section, mentioning the limitations of our study (lines 203-214):

While our study shows that 2’-O-Me levels can distinguish different types of cellular growth arrest, it has certain limitations. We exclusively focused on 2’-O-Me, representing only one type and slightly less than half of all rRNA modifications. Moreover, only a few 2’-O-Me showed plasticity. Thus, including Ψ and base modifications in future experiments will likely improve the classification of the different cellular states and identify additional sites specifically associated with cell proliferation and senescence. Similarly, we concentrated on HDF and one inducer of cellular senescence and quiescence each. Further studies will be required to determine if the discovered mechanisms are universal and if the same sites are differentially modified in other cell types, in oncogene-induced or replicative senescence, and in serum-starvation-induced quiescence. Finally, whether the observed changes in 2’-O-Me patterns and the shift in ribosome processing pathways play an active part in regulating proliferation and senescence, for instance, by modulating specific mRNA translation, still remains to be tested.

b) The authors do not really discuss whether there is any visible pattern when it comes to the 2D/3D positioning of the altered rRNA modifications and importantly how these changes may directly influence ribosome function.

Thank you for this suggestion. We mapped all 2’-O-Me positions on the ribosomal RNAs and highlighted the nine differentially modified sites in a new figure (Fig. 1E). We describe these findings in the text (line 119-123):

Mapping the nine differently methylated sites to the 3D structure of the human 80S ribosome (Fig. 1E) revealed that they were close to functional regions, including the intersubunit bridges, the polypeptide exit tunnel, and the body and head of the SSU (Fig. 1E). However, positions in the vicinity of the peptidyl transferase center (PTC) were not variable, indicating their essential role in core ribosome function.

© 2024. Published by The Company of Biologists under the terms of the Creative Commons Attribution License (https://creativecommons.org/licenses/by/4.0/).

Moreover, we discuss the possible consequences of these modifications for ribosome function (line 175- 183):

The ribosome's catalytic core, the PTC, appears to be protected from changes because most 2'-O-Me positions surrounding the PTC remained almost fully methylated. In contrast, SSU-C797 and G867, both hypo-methylated in P cells, are close to each other in the body of the SSU in the vicinity of bridge eB12, which consists of a salt bridge between eL19 and SSU-G909 and G910 (Kisly et al., 2016). LSU- C1303 is near uL4, contributing to the second constriction site of the polypeptide exit tunnel (Dao Duc et al., 2019). LSU-G3723 is near the interaction layer of the SSU and LSU, the decoding center, and the B2a intersubunit bridge. This region is particularly interesting because it undergoes significant rearrangements to fine-tune the rotation of the ribosome during translation (Lomakin et al., 2017; Sulima et al., 2014).

c) The title is a bit misleading in the sense that it almost implicitly reads somewhat like large changes of methylation at multiple positions is occurring. Moreover, the title should be more precise and less general, as well, since the analysis covers 2'O methylations, and not the other known types of rRNA methylations, and to integrate (as mentioned above) the paucity of the observed alterations should be made clearer.

We agree with the reviewer's comment and changed the title accordingly (line 1-2):

2'-O-ribose methylation levels of ribosomal RNA can distinguish different types of growth arrest in human dermal fibroblasts

We further specified the description of the observed changes by modifying the abstract as follows (line 33-35):

We compared both modes of cell cycle arrest to proliferating cells and could indeed distinguish these conditions by their overall 2'-O-Me patterns. Methylation levels at a small fraction of sites showed plasticity and correlated with the expression of specific snoRNAs but not fibrillarin.

Moreover, as explained above, we added a paragraph to the discussion, indicating the limitations of our study (line 203-214).

2) Effect of rRNA methylation on cellular proliferation:

a) Are there any reasons to show only the HDF76 SNORD87/88 knockdown in Figure 3 and the average of the three HDF knockdown in supplementary Figure 4? It would be critical to show how the HDF samples behave individually considering the underlying putative biological heterogeneity.

Thank you very much for this suggestion. We now show the individual growth curves of HDF85, HDF161, and an additional independent replicate of HDF76 in Figure S4A-C. Knockdown of SNORD88 reduced proliferation in cells from all three donors, while knockdown of SNORD87 boosted proliferation only in HDF76 (four independent replicates) and HDF85, but not in HDF161. We discuss this finding in the text (line 190-193):

Our results indicate that even small changes in snoRNA levels might directly affect the proliferative status of cells. A knockdown of SNORD87, guiding a site hypomethylated in proliferating cells, was sufficient to increase cell proliferation in two out of three tissue donors and reduce the fraction of S- phase cells, suggesting a faster progression through the cell cycle.

Moreover, we ensured that the individual cell donors were clearly indicated in all figures, using shapes for the data points.

b) SNORD87 knockdown has no effect on proliferation in NIH3T3 or HeLa cells which is somehow in contradiction with the conclusions of the authors. The authors should comment on that.

© 2024. Published by The Company of Biologists under the terms of the Creative Commons Attribution License (https://creativecommons.org/licenses/by/4.0/).

Since we observed that SNORD87 depletion does not boost global protein synthesis (new Fig. 4E), we speculate that it might alternatively promote proliferation by affecting the translation of specific mRNAs or translational fidelity. Since immortalized cell lines already show increased proliferation, they might be insensitive to these additional stimuli. Due to the biological differences between normal and cancer cells, we focused the revised version of our study exclusively on HDF. We consequently removed all data from immortal cell lines (HeLa, NIH3T3).

c) Based on the author's conclusions, one could expect that SNORD87 knockdown should delay entrance into senescence or alternatively increasing SNORD87 (and the resulting 2'O methylation) may have a positive effect on cell proliferation? Same should be equally true for SNORD127? It is also not fully clear why the authors only focus on Hela cells in this case (which was unaffected with SNORD87). What about NIH3T3 and/or the HDF donor cells? These types of experiments would strongly strengthen the proposed direct effect of these rRNA modifications alteration on the regulation of cell proliferation.

We agree that it would be interesting to investigate the consequences of SNORD87 or SNORD127 depletion on cellular senescence induction, as we would expect a delayed onset of senescence. However, this experiment would require generating snoRNA mutated and control CRISPR/Cas9 edited HDF clones, which is not trivial and not yet established in our laboratory. Studying senescence in cancer cell lines, such as HeLa, is of limited biological relevance, and snoRNA depletion by ASOs in HDF would require repeated transfections over two weeks while the cells are treated with H_2O_2, which likely imposes additional stress during senescence induction, and will therefore be challenging to establish and control. Thus, although the reviewer's suggestion is excellent, we believe that the experiment is not feasible within the scope of this study.

Reviewer 3

The study aims to better understand the potential role of rRNA 2'-O-ribose methylations, notably during cell senescence. It is part of an emerging field of research that aims to describe the extent to which ribosomes can be heterogeneous. Using RiboMeth-seq, the authors profiled 2'-O-methylations in rRNA of proliferating vs senescent vs quiescent human cells. This made it possible to reveal changes in the methylation levels at a very few sites rRNA sites (n=9). The authors also noticed some alterations in pre-rRNA processing. Of note, methylation levels at some differentially-methylated rRNA sites correlate with expression of the cognate SNORD. Finally, SNORD loss-of-function experiments are accompanied by altered cell proliferation. To my knowledge, this is one of the first, if not the first, study of rRNA methylation profiles in senescent cells. This type of study suggests that changes in rRNA methylation are dependent on the quiescence/senescence/proliferation state.

I find this short manuscript interesting but before considering its publication, I would like the authors to clear up some ambiguities. In my view, some statements are over-interpreted and deserve to be nuanced in order to be more in line with the observed results. Addressing #3 and #8 seems to me to be a priority. I am not an English speaker and I would not allow myself to make relevant comments.

Thank you very much for the accurate summary and for considering our study interesting.

#1 - In my view, the title is somewhat ambiguous. Based on such a title and the abstract, one could easily interpreter that the authors have identified rRNA sites whose methylation changes are specifically associated with the senescence state. As I understand it, the situation is more complex. As stated by the authors: "The SSU-C1391, G1447, and LSU-C3680, G3723 methylation in SIPS differed from P or Q cells suggesting a specific association with SIPS, however the biological variance between donors at these sites precludes a conclusion from the current data". I therefore invite the authors to qualify their statement. If, on the other hand, the authors believe that they have truly identified senescence-specific rRNA methylations, then these latter should be highlighted more explicitly and discussed accordingly.

We fully agree with the reviewer. To address this suggestion, we refined our statistical analysis using the Qlucore software, added two new t-SNE blots to show the clustering of samples before

© 2024. Published by The Company of Biologists under the terms of the Creative Commons Attribution License (https://creativecommons.org/licenses/by/4.0/).

and after adjustment for donor variation (Figure 1A, B), and thereby now also identified three sites specifically associated with cellular senescence. To better explain and qualify our findings, we rewrote the respective passage of the text (lines 108-118):

Next, we inspected individual 2'-O-Me sites on the small (SSU) and large ribosomal subunit (LSU) potentially underlying these global differences (Fig. 1C,D, and Tables S2-S4). While the majority of sites were highly methylated (RMS > 0.9), nine positions that were not fully methylated showed significant differences between the cellular states (SSU: G436, C797, G867, C1272; LSU: G1303, A2388, G2411, G3723, G4588; adjusted multi-factor ANOVA q < 0.1). In line with our previous observations (Fig. 1B), the most pronounced differences were again between P and the other two growth states (SSU: G436, C797, G867, C1272; LSU: G1303, G2411, G3723, G4588; Tukey p < 0.05, Table S2). Still, three sites also significantly differed between Q and SIPS (LSU: A2388, G3723, G4588; Tukey p < 0.05, Table S2), suggesting their specific association with cellular senescence. Interestingly, the average RMS scores in all sites were slightly higher in Q and SIPS than in P cells, with altered methylation sites predominantly occurring at G/C nucleotides.

#2 - "In summary, 2'-O-Me profiling revealed a distinct pattern between senescent, quiescent and proliferating cells, consistent with the variable sites identified recently in cancer cells (Marcel et al., 2020)". It would be interesting if the authors would give more information on these differentially- methylated rRNA sites. Are they sites known to be variable in other biological systems? Are they altered in cancers? It seems to me that it would also be reasonable for the authors to state clearly that the observed changes are relatively minor both in terms of the number of sites and the magnitude of the changes. I also get the impression that the changes are mostly in rRNA sites that are never 95-100% methylated in any of the cell types analyzed. This should also be mentioned and discussed accordingly. Such a comparison could be given in the supplementary data.

Thank you very much for this suggestion; we agree that such information would be valuable to the reader of our manuscript. Therefore, we generated a heatmap summarizing our and other studies that analyzed 2'-O-Me patterns (Figure S7). We also added the following text to the discussion (line 184-189):

Strikingly, several sites we identified to be hypomodified in P cells also showed lower modification levels in developing compared to adult mouse tissues (Häfner et al., 2023; Hebras et al., 2020), in human embryonic stem cells compared to differentiated ectoderm (Häfner et al., 2023), in diffuse large B-cell lymphoma compared to reactive lymph nodes (Krogh et al., 2020), and in breast cancer cell lines (Marcel et al., 2020), suggesting their inverse correlation with cell proliferation, cancer, and pluripotency (Fig. S7).

#3 - "Even small methylation changes were accompanied by comparable alterations in the snoRNAs levels (Figure 1b, d; 61 Supplementary Figure 3), suggesting that the observed hypomethylation might be due to the limiting levels of the snoRNAs". As I understand it, the measurements of SNORD expression by Northern are not consistent with those made by RT-qPCR (Figure 1d), especially when comparing proliferating vs quiescent cells. Could the authors provide explanations for such discrepancies? What about the expression levels of SNORD host-genes transcripts? In my view, this could be easily addressed.

We agree with the reviewer and addressed this issue by re-analyzing two previous mRNA and small RNA datasets that were generated using the same senescence model by our group. The caveat of this approach is that the datasets only included the comparison of quiescent and senescent but not proliferating fibroblasts. We now present the results in Fig. S4 and noticed that the small RNA sequencing of the snoRNAs matched closer to the methylation levels and the northern blots than the qRT-PCR data. In some but not all cases, the host gene expression was similar to the corresponding snoRNA. Accordingly, we added the following sentence to the manuscript (line 132-133):

#The snoRNA expression correlated in some, but not in all cases, with the respective host gene (Fig. S5).

© 2024. Published by The Company of Biologists under the terms of the Creative Commons Attribution License (https://creativecommons.org/licenses/by/4.0/).

#4 - "These results indicate that the changes in methylation and snoRNA expression between P and SIPS/Q cells are not just a side-effect but are more intrinsically linked to cell proliferation". Because the authors did not measure rRNA methylation levels in SNORD-depleted cells, this conclusion is, in my view, over-interpreted and should be modified accordingly.

We agree and toned the respective passage down to avoid over-interpretation (line 162-164):

Taken together, these results indicate that the changes in snoRNA expression between P and SIPS/Q cells are not just a side-effect but are more intrinsically linked to cell proliferation.

#5 - Figure 3_ panel a - As I understand it, control cells have also been transfected with a non-targeting oligo. If this is the case, then it should be stated more explicitly in the figure and its legend.

We added the following clarification to the legend of Fig. 4:

HDF76 were transfected with ASOs specifically targeting SNORD87 or SNORD88C, as well as a non-targeting scrambled control ASO (control).

#6 - Figure 3_ panel b - I am not sure I understand the rationale for mutating SNORD127 in HeLa cells. Indeed the corresponding site (SSU-G1147) is methylated up to 70% in HeLa cells (doi: 10.1093/nar/gkw482) whereas the authors find it largely under-methylated, not to say non-methylated in this study. In this regard, how sure are the authors that a RMS score around 0-30 is not simply technical background noise?

We agree with the reviewer. We realized that immortalized cancer cell-lines are not comparable to primary cells and thus do not represent an appropriate model to evaluate senescent phenotypes. In addition, HeLa cells, perhaps due to their polyploidy, can also behave rather heterogeneously between lines from different labs. We therefore focused our revised manuscript exclusively on primary human fibroblasts. Thus, we removed all data from HeLa and other immortalized cell lines.

#7 - "A cut between the T and A three nucleotides upstream of the D-box motif (CUGA) was inserted in SNORD127 (Supplementary Figure 5a,b)". As I understand it, the authors only modified the antisense element of SNORD127 via CRISPR-Cas9. If this is the case, I do not understand why the expression level of SNORD127 is reduced. This is an unexpected result since only the 5'-3' terminal stem structure is required for efficient processing and accumulation. Can the authors comment? I also invite the authors to draw the WT and mutated antisense segments of SNORD127 in Figure 3. This will help the reader.

Thank you for this comment. We agree that the reduced levels of SNORD127 were surprising and challenging to explain. As indicated in the next comment (#8), this finding could represent an artifact from the clonal selection because only one clone was analyzed. In addition, the polyploid state of HeLa cells complicates CRISPR/Cas9-based gene editing, and cancer cell lines are generally difficult to compare to primary cells. Therefore, we now exclusively focused on HDF and removed all data from HeLa and other cell lines.

#8 - "Two days after transduction, mKate2- positive cells were isolated by FACS. After antibiotic selection with blasticidin and puromycin for two weeks, dilution cloning was performed in 96-well plates, single clones were picked, and analyzed by Sanger-sequencing (Eurofins)". How many independent and randomly-picked SNORD127M clones did the authors analyse when they measured cell growth (Figure 3 panels b,d)? Moreover, were the control cells also transduced with irrelevant constructs and underwent the same dilution cloning procedure? In my view, the use of matched control clones is essential to avoid bias and other artefacts. This is all the more important as the analysis of mutated Hela cells is very minimalist. Indeed, one would have expected such studies to be accompanied by analyses of cell cycle and apoptosis. Finally, and as stated above, the authors did not profile rRNA methylation in SNORD127M HeLa cells. Perhaps primer extension could be made with low dNTPs concentration.

We agree and are very grateful for the insightful suggestions. As mentioned above, we removed

© 2024. Published by The Company of Biologists under the terms of the Creative Commons Attribution License (https://creativecommons.org/licenses/by/4.0/).

the HeLa data from the manuscript. In future studies, we will attempt to gene-edit primary cells or cell lines with a stable diploid genome using the described strategies, including the suggested controls and validation of reduced methylation by primer extension or direct RNA Nanopore sequencing. We believe that these experiments will provide valuable mechanistic data on snoRNA function.

However, we followed the reviewer's suggestion and now included an analysis of the cell cycle, apoptosis, and global protein synthesis in Fig. 4C-E to provide a more thorough characterization of HDF physiology after SNORD87 and SNORD88 depletion. We describe our findings as follows (line 158- 162):

Cell cycle analysis by HOECHST33342 staining revealed that SNORD88A/B/C depletion increased the fraction of cells in the S phase and decreased cells in the G1/G0 phase, while transfection with the SNORD87 ASO showed the opposite effect (Figure 4C, S6A). Surprisingly, we did not detect differences in apoptosis (Figure 4D, S6B) or global protein synthesis (Figure 4E), which might explain these observations.

Second decision letter

MS ID#: JOCES/2023/261930

MS TITLE: 2'-O-ribose methylation levels of ribosomal RNA can distinguish different types of growth arrest in human dermal fibroblasts

AUTHORS: Guohuan Yang, Maximilian Schmid-Siegel, Clemens Heissenberger, Isabelle Kos-Braun, Martina Prechtl, Gabriel Meca-Laguna, Anja Wagner-Schrittwieser, Vera Pils, Barbara Meixner, Koray Tav, Markus Hengstschlaeger, Johannes Grillari, Martin Kos, and Markus Schosserer

ARTICLE TYPE: Short Report

Based on the revisions to your earlier submission I am happy to tell you that your manuscript has been accepted for publication in Journal of Cell Science, pending standard ethics checks.

© 2024. Published by The Company of Biologists under the terms of the Creative Commons Attribution License (https://creativecommons.org/licenses/by/4.0/).
